# Oxygen targets and 6-month outcome after out of hospital cardiac arrest: a pre-planned sub-analysis of the targeted hypothermia versus targeted normothermia after Out-of-Hospital Cardiac Arrest (TTM2) trial

**DOI:** 10.1186/s13054-022-04186-8

**Published:** 2022-10-21

**Authors:** Chiara Robba, Rafael Badenes, Denise Battaglini, Lorenzo Ball, Filippo Sanfilippo, Iole Brunetti, Janus Christian Jakobsen, Gisela Lilja, Hans Friberg, Pedro David Wendel-Garcia, Paul J. Young, Glenn Eastwood, Michelle S. Chew, Johan Unden, Matthew Thomas, Michael Joannidis, Alistair Nichol, Andreas Lundin, Jacob Hollenberg, Naomi Hammond, Manoj Saxena, Annborn Martin, Miroslav Solar, Fabio Silvio Taccone, Josef Dankiewicz, Niklas Nielsen, Anders Morten Grejs, Florian Ebner, Paolo Pelosi, TTM2 Trial collaborators, Jan Bělohlávek, Clifton Callaway, Alain Cariou, Tobias Cronberg, David Erlinge, Jan Hovdenes, Hans Kirkegaard, Helena Levin, Matt P. G. Morgan, Per Nordberg, Mauro Oddo, Christian Rylander, Christian Storm, Susann Ullén, Matt P. Wise, Kathy Rowan, David Harrison, Paul Mouncey, Manu Shankar-Hari, Duncan Young, Theis Lange, Karolina Palmér, Ulla-Britt Karlsson, Simon Heissler, Frances Bass, John Myburgh, Colman Taylor, Adele Bellino, Marwa Abel-all, Ben Finfer, Carolyn Koch, Yang Li, Anne O’Connor, Julia Pilowsky, Tina Schneider, Anna Tippett, Bridget Ady, Tessa Broadley, Amanda Brown, Liz Melgaard, Mimi Morgan, Vanessa Singh, Rebecca Symons, Kathrin Becker, Nathalie Sante, Vendula Saleova, Silvie Zerzanova, Samia Sefir-Kribel, Ute Lübeck, Martina Carrara, Kathryn Fernando, Diane Mackle, Leanlove Navarra, Judith Riley, Elin Westerheim, Marianne Flatebø, Ameldina Ceric, Zana Haxhija, Lovisa Terling, Lena Bossmar, Liz Jergle, Helén Holm Månsson, Samia Abed Maillard, Andreja Vujicic Zagar, Christina Jodlauk, Jennifer Scrivens, Kate Ainscough, Ciara Fahey, Rinaldo Bellomo, Leah Peck, Helen Young, Winston Cheung, Rosalba Cross, Michael Hayes, Nitin Jain, Mark Kol, Asim Shah, Atul Wagh, Helen Wong, F. Eduardo Martinez, Gail Brinkerhoff, Dustin Bush, Antony Stewart, Anders Aneman, Lien Lombardo, Peter McCanny, James Penketh, Ian Seppelt, Rebecca Gresham, Julie Lowrey, Kristy Masters, Christina Whitehead, James Walsham, Meg Harward, Josephine Mackay, Jason Meyer, Emma Saylor, Ellen Venz, Krista Wetzig, Wade Stedman, Angela Ashelford, Sharon Mar, Miyuki Tokumitsu, Elizabeth Yarad, Hergen Buscher, Claire Reynolds, Andrew Udy, Aidan Burrell, Jasmin Collins, Dashiell Gantner, Victoria Emma-Leah Martin, Phoebe Mccracken, Vinodh Nanjayya, Alexander Sacha Richardson, Meredith Young, Angaj Ghosh, Simone Said, Ronny Beer, Frank Hartig, Raimund Helbok, Sebastian Klein, Andreas Peer, Jacques Creteur, Dominique Durand, Matthias Dupont, Sigrid Christiaens, Carola Claes, Sebastiaan Deckx, Bert Ferdinande, Sanne Lenaerts, Wilifred Mullens, Sarah Stroobants, Evi Theunissen, David Verhaert, Ondřej Šmíd, Marek Flaksa, David Kemlink, Jan Malík, Michal Otáhal, Jan Rulíšek, Michal Šíranec, Zdeněk Stach, Anna Valeriánová, Petra Zavadilova, Miroslav Solař, Róber Bánszky, Jana Červená, Renata Černá Pařízková, Libor Šimůnek, Filip Varhaník, Jiří Karásek, Matěj Strýček, Anders Grejs, Steffen Christensen, Peter Juhl-Olsen, Ida Katrine Thomsen, Lisa Gregersen Østergaard, Albert Cao, Pierre Dupland, Ariane Gavaud, Paul Jaubert, Mathieu Jozwiak, Nathalie Marin, Guillaume Savary, Nicolas Deye, Bruno Megarbane, Pierre Mora, Laetitia Sutterlin, Stephane Legriel, Hugo Bellut, Alexis Ferre, Guillaume Lacave, Marine Paul, Jean-Baptiste Lascarrou, Emmanuel Canet, Charlotte Garret, Arnaud Felix Miaihle, Jean Reignier, Philippe Vignon, Thomas Daix, Arnaud Desachy, Bruno Evrard, Bruno Francois, Anne-Laure Fedou, Marine Goudelin, Christoph Leithner, Jens Nee, Kaspar Josche Streitberger, Giulia Bonatti, Iacopo Firpo, Paolo Frisoni, Arianna Iachi, Simona Maiani, Maura Mandelli, Fabio Tarantino, Alberto Barbieri, Elisabetta Bertellini, Enrico Giuliani, Gabriele Melegari, Erik Roman-Pognuz, Giorgio Berlot, Umberto Lucangelo, Elisabetta Macchini, Vibeke Aune, Tomas Drægni, Simon Jacobsen, Søren Pieschke, Åse Rasmussen, Gro Ringstad Akselsen, Halvor Langeland, Daniel Bergum, Therese M Erbe, Pål Klepstad, Helle M Næss, Roy Bjørkholt Olsen, Lena Eriksen Skjelnes, Marius Holen, Joakim Iver Post, Rune Fanebust, Linda Hårteig Sørensen, Ken Åge Kårstad, CarstenFredrik Wickman, Colin Barnes, Ben Barry, Nina Beehre, Dick Dinsdale, Sam Edney, Anna Hunt, Harriet Judd, Charlotte Latimer-Bell, Cassie Lawrence, James Moore, Shaanti Olatunji, Alex Psirides, Chelsea Robinson, Kate Tietjens, Jason Wright, David Knight, Brandon Birker, David Bowie, Tara Burke, David Closey, Rosalind Crombie, Neil Davidson, Seton Henderson, Louise Hitchings, James McKay, Jan Mehrtens, Emmeline Minto, Stacey Morgan, Anna Morris, Jay Ritzemar-Carter, Jessica Roberts, Geoffrey Shaw, Katherine Townend, Kymbalee Vander Heyden, Marita Ahlqvist, Roman Desta Lindgren, Ingrid Eiving, Patrik Martner, Elisabeth Myhrman, Birgitta Ryding, Joachim Düring, Mattias Bergström, Mattias Bohm, Ingrid Didriksson, Petrea Frid, Katarina Heimburg, Marina Larsson, Oscar Lundberg, Stefan Olsson Hau, Simon Schmidbauer, Ola Borgquist, Anne Adolfsson, Anna Bjärnroos, Erik Blennow-Nordström, Irina Dragancea, Thomas Kander, Anna Lybeck, Gustav Mattiasson, Olof Persson, Malin Rundgren, Susann Schrey, Erik Westhall, Martin Annborn, Sara Andertun, Nerida Gustavsson, Lisa Hassel, Jesper Johnsson, Marie Nelderup, Heléne Petersson, Jörgen Petersson, Frideriki Stafilidou, Johan Undén, Frida Antonsson, Git Bergman, Jörgen Gamroth, Maria Meirik, Katarina Rudolfsson, Helena Sandberg, Martin Thorsson, Kristin Savolainen, Maria Hansbo, Malin Helliksson, Björne Nödtveidt, Johan Sanner, Victoria Sem, Camilla Sund Lindquist, Akil Awad, Anna-Sofia Börjesson, Malin Hedberg, Mia Henning, Per Petersen, Emelia Dahlberg, Johan Forshammar, Veronica Svensson, Michael Wanecek, Håkan Eskilsson, Daniel Rodriguez-Santos, Åsa Appelqvist, Henrietta Jidbratt, Elisabeth Johansson, Lars Kiszakiewicz, Åsa Nilsson, Sinnika Olsson, Anders Paulsson, Urszula Stempel, Andreas Thoren, Stefan Persson, Ida Berglund, Eric Bergström, Cathrine Törnqvist, Ingela Östman, Sten Rubertsson, Ing-Marie Larsson, Elin Söderman, Ewa Wallin, Joanna Wessbergh, Thomas Halliday, Filippa Engvall, Nawfel Ben-Hamouda, Adriano Bernini, Pierre-Nicolas Carron, Philippe Eckert, Eva Favre, John-Paul Miroz, Paola Morelli, Olivier Muller, Jan Novi, Andrea Rosseti, Madeleine Schnorf, Matthias Haenggi, Anja Levis, Sandra Nansoz, Marianne Roth & Team, Nicole Söll, Claudia Schrag, Mensur Alicajic, Philipp Baier, Joel Dütschler, Dominique Flügel, Edith Fässler, Ruth Gamio-Veis, Marc Güpfert, Yvonne Hilpertshauser, Stefan Hägele-Link, Gian-Reto Kleger, Peter Krähenmann, Maria Elisabeth Mair, Nadja Schai, Christoph Strohmaier, Peter Tangl, Dominik Zieglgänsberger, Marco Maggiorini, Gabriele Claus, Gabi Consani-Vogel, Lukas Imbach, Samira Kaiser, Eva-Maria Kleinert, Pedro David Wendel Garcia, Tiziano Cassina, Pamela Agazzi, Bruno Capelli, Gabriele Casso, Martino Regazzi, Hervé Schlotterbeck, Gabriele Via, Michele Villa, Jenny Brooks, Eve Cocks, Jade Cole, Jacqueline Curtin, Michelle Davies, Rhys Davies, Stephen Fernandez, Julie Highfield, Helen Hill, Lydia Pennant, Sofia Rose, Emma Thomas, Angharad Williams, Peter McGuigan, Stephen Haffey, Aisling O’Neill, Kathryn Ward, Jeremy Bewley, Anna Chillingworth, Julie Cloake, Libby Cole, Hilary Galvin, Zoe Garland, Lisa Grimmer, Bethany Gumbrill, Lucy Howie, Rebekah Johnson, Chloe Searles, Agnieszka Skorko, Katie Sweet, Victoria Taylor, Denise Webster, Thomas Keeble, Gill Adams, Rajesh K. Aggarwal, Jo-Anne Cartwright, Steven Church, Gerald J. Clesham, John R. Davies, Kelly Farrell, Reto Gamma, Jane Harding, Rohan Jagathesan, Alamgir Kabir, Paul A. Kelly, Lauren Kittridge, Maria Maccaroni, Gracie Maloney, Marco Mion, Naveen Nain, Raghunath Nalgirkar, Gyanesh Namjoshi, Stacey Pepper, Emily Redman, Jeremy Sayer, Amanda Solesbury, Kare H. Tang, Sali Urovi, Kunal Waghmare, Noel Watson, Teresa Webber, Peter Isherwood, Conor Bentley, Colin Bergin, Ronald Carrera, Amy Clark, Lauren Cooper, Liesl Despy, Natalie Dooley, Karen Ellis, Emma Fellows, Stephanie Goundry, Samantha Harkett, Christopher McGhee, Aoife Neal, Hazel Smith, Catherine Snelson, Elaine Spruce, Tony Whitehouse, Kamal Yakoub, Andrew Walden, Shauna Bartley, Parminder Bhuie, Matthew Frise, Nicola Jacques, Liza Keating, David Pogson, Zoe Daly, Steve Rose, Jonathan Bannard-Smith, Rachael Quayle, Nigel Chee, Nina Barratt, Katie Bowman, Debbie Branney, Elizabeth Howe, Maria Letts, Sally Pitts, Luke Vamplew, Clifton W. Callaway, Sara Difiore Sprouse, Ankur A. Doshi, Jennifer Fugate, Amy M. Headlee, Eelco F. M. Wijdicks

**Affiliations:** 1 Anesthesia and Critical Care, San Martino Policlinico Hospital, IRCCS for Oncology and Neuroscience, Genoa, Italy; 2 Department of Surgical Sciences and Integrated Diagnostics, University of Genoa, Viale Benedetto XV 16, Genoa, Italy; 3 Department of Anesthesiology and Surgical-Trauma Intensive Care, Hospital Clínic Universitari de Valencia, Valencia, Spain; 4 Department of Surgery, University of Valencia, Valencia, Spain; 5 Department of Medicine, University of Barcelona, Barcelona, Spain; 6 Department of Anaesthesia and Intensive Care, A.O.U. “Policlinico-San Marco”, Catania, Italy; 7 Copenhagen Trial Unit, Centre for Clinical Intervention Research, Copenhagen University Hospital - Rigshospitalet, Copenhagen, Denmark; 8 Department of Regional Health Research, Faculty of Health Sciences, University of Southern Denmark, Odense, Denmark; 9 Department of Clinical Sciences Lund, Neurology, Skåne University Hospital, Lund University, Getingevägen 4, 222 41 Lund, Malmö, Sweden; 10 Department of Clinical Sciences Lund, Anesthesia and Intensive Care, Lund University, Lund, Sweden; 11 Institute of Intensive Care Medicine, University Hospital of Zurich, Rämistrasse 100, 8091 Zurich, Switzerland; 12 Medical Research Institute of New Zealand, Private Bag 7902, Wellington, 6242 New Zealand; 13 Intensive Care Unit, Wellington Regional Hospital, Wellington, New Zealand; 14 Australian and New Zealand Intensive Care Research Centre, Department of Epidemiology and Preventive Medicine, School of Public Health and Preventive Medicine, Monash University, Melbourne, VIC Australia; 15 Department of Critical Care, University of Melbourne, Parkville, VIC Australia; 16 Department of Intensive Care, Austin Hospital, Melbourne, Australia; 17 Department of Anaesthesia and Intensive Care, Biomedical and Clinical Sciences, Linköping University, Linköping, Sweden; 18 Department of Clinical Sciences Malmö, Lund University, Malmö, Sweden; 19 Department of Operation and Intensive Care, Hallands Hospital Halmstad, Lund University, Halland, Sweden; 20 University Hospitals Bristol NHS Foundation Trust, Bristol, UK; 21 Division of Intensive Care and Emergency Medicine, Department of Internal Medicine, Medical University Innsbruck, Innsbruck, Austria; 22 Monash University, Melbourne, VIC Australia; 23 Department of Anaesthesiology and Intensive Care Medicine, Institute of Clinical Sciences, Sahlgrenska Academy, University of Gothenburg, 423 45 Gothenburg, Sweden; 24 Department of Clinical Science and Education, Södersjukhuset, Centre for Resuscitation Science, Karolinska Institutet, Solna, Sweden; 25 Malcolm Fisher Department of Intensive Care, Royal North Shore Hospital, Critical Care Division, The George Institute for Global Health, Faculty of Medicine, UNSW Sydney, Sydney, Australia; 26 Intensive Care Unit, St George Hospital, Sydney, Australia; 27 Department of Clinical Medicine, Anaesthesiology and Intensive Care, Lund University, Lund, Sweden; 28 Department of Internal Medicine, Faculty of Medicine in Hradec Králové, Charles University, Hradec Králové, Czech Republic; 29 Department of Internal Medicine - Cardioangiology, University Hospital Hradec Králové, Hradec Králové, Czech Republic; 30 Department of Intensive Care Medicine, Université Libre de Bruxelles, Hopital Erasme, Brussels, Belgium; 31 Department of Clinical Sciences Lund, Cardiology, Skåne University Hospital, Lund University, Lund, Sweden; 32 Department of Clinical Sciences Lund, Anaesthesia and Intensive Care and Clinical Sciences Helsingborg, Helsingborg Hospital, Lund University, Lund, Sweden; 33 Department of Intensive Care Medicine, Aarhus University Hospital, Aarhus, Denmark; 34 Department of Clinical Medicine, Aarhus University, Aarhus, Denmark; 35 Department of Clinical Sciences Lund, Anesthesia and Intensive Care, Helsingborg Hospital, Lund University, 251 87 Helsingborg, Sweden

**Keywords:** Cardiac arrest, Hypoxemia, Hyperoxemia, Mortality, Neurological outcome

## Abstract

**Background:**

Optimal oxygen targets in patients resuscitated after cardiac arrest are uncertain. The primary aim of this study was to describe the values of partial pressure of oxygen values (PaO_2_) and the episodes of hypoxemia and hyperoxemia occurring within the first 72 h of mechanical ventilation in out of hospital cardiac arrest (OHCA) patients. The secondary aim was to evaluate the association of PaO_2_ with patients’ outcome.

**Methods:**

Preplanned secondary analysis of the targeted hypothermia versus targeted normothermia after OHCA (TTM2) trial. Arterial blood gases values were collected from randomization every 4 h for the first 32 h, and then, every 8 h until day 3. Hypoxemia was defined as PaO_2_ < 60 mmHg and severe hyperoxemia as PaO_2_ > 300 mmHg. Mortality and poor neurological outcome (defined according to modified Rankin scale) were collected at 6 months.

**Results:**

1418 patients were included in the analysis. The mean age was 64 ± 14 years, and 292 patients (20.6%) were female. 24.9% of patients had at least one episode of hypoxemia, and 7.6% of patients had at least one episode of severe hyperoxemia. Both hypoxemia and hyperoxemia were independently associated with 6-month mortality, but not with poor neurological outcome. The best cutoff point associated with 6-month mortality for hypoxemia was 69 mmHg (Risk Ratio, RR = 1.009, 95% CI 0.93–1.09), and for hyperoxemia was 195 mmHg (RR = 1.006, 95% CI 0.95–1.06). The time exposure, i.e., the area under the curve (PaO_2_-AUC), for hyperoxemia was significantly associated with mortality (*p* = 0.003).

**Conclusions:**

In OHCA patients, both hypoxemia and hyperoxemia are associated with 6-months mortality, with an effect mediated by the timing exposure to high values of oxygen. Precise titration of oxygen levels should be considered in this group of patients.

*Trial registration*: clinicaltrials.gov NCT02908308, Registered September 20, 2016.

**Supplementary Information:**

The online version contains supplementary material available at 10.1186/s13054-022-04186-8.

## Background

Cardiac arrest is a major cause of mortality and morbidity, and over the last years [1, 2], attention has risen toward the levels of oxygenation to achieve as an essential determinant of secondary brain injury and worsened outcomes [3]. Mechanical ventilation is commonly required to avoid hypoxemia [4], which is a well-known cause of anoxic brain injury promoting secondary brain and reperfusion-related damage [5, 6]. Recent literature has also focused on the role of hyperoxemia in critically ill patients [7–10]. Supplemental oxygen can correct hypoxemia, thereby supporting cell function, metabolism, and limiting organ dysfunction. However, it might have detrimental effects on patients’ outcomes through different pathophysiological mechanisms, such as the production of reactive oxygen species and free radicals yielding secondary damage due to reperfusion injury [11–17]. Studies exploring the role of hypo- and hyperoxemia after cardiac arrest are not conclusive. They present several heterogeneities in terms of study design, sample size and outcome definition, as well as inconsistent results, especially when compared to the preclinical cardiac arrest models [8, 18–21].

We therefore performed a secondary analysis of the Targeted Hypothermia versus Targeted Normothermia after Out-of-Hospital Cardiac Arrest (TTM2) trial that included Out-of-Hospital Cardiac Arrest (OHCA) patients. The aim was to assess the oxygen targets, the incidence of episodes of hypoxemia and hyperoxemia in the first 72 h of mechanical ventilation, and their association with patients’ 6-months outcome (mortality and neurological status).

## Methods

This was a pre-planned secondary analysis of the TTM2 trial, which was an international, multicenter randomized controlled trial comparing the effects of normothermia (temperature ≤ 37.5 °C), versus hypothermia (target 33 °C until 28 h after randomization, and then rewarming to 37 °C) [22, 23]. This sub-analysis was conducted according to the Strengthening the Reporting of Observational Studies in Epidemiology (STROBE) reporting guidelines [24] (Additional file 1: Table S1). Ethical approval was obtained in the coordinating center and in each participating center as well as informed consent according to local regulations. This sub-study was conducted in accordance with the principles of the Declaration of Helsinki, and the Medical Research Involving Human Subjects Act (WMO) and was approved on the 23^rd^ of February 2017 by the TTM2 steering committee (https://ttm2trial.org/substudy-proposals). The protocol of the analysis was published [22]. No further ethical approval was necessary for the development of this study.

### Objectives and definitions

The primary aim was to describe the arterial partial pressure of oxygen (PaO_2_) values observed in OHCA patients in the first 72 h of mechanical ventilation and the occurrence of episodes of hypo/hyperoxemia. As previous studies have considered arterial oxygen thresholds of < 60 mmHg and > 300 mmHg when evaluating associations between oxygen exposure and outcome [7, 8, 18, 25], we pre-specified that we would initially evaluate the same thresholds, and then, we aimed to calculate the “best” threshold of hypo/hyperoxemia associated with poor outcome. For primary analysis, three patients’ groups according to conventional thresholds were defined: (1) hypoxemia with one or more episodes of PaO_2_ levels < 60 mmHg; (2) normoxemia (including mild-moderate hyperoxemia, with PaO_2_ levels between 60 and 300 mmHg; and (3) severe hyperoxemia with one or more PaO_2_ levels > 300 mmHg. The secondary objectives were to assess: (1) the association between hypo/hyperoxemia during the first 72 h of mechanical ventilation with mortality and neurological outcome at 6-months; (2) the best threshold of hypo/hyperoxemia associated with mortality and poor neurological outcome; (3) the cumulative effect of the “dose” (oxygen exposure over time, PaO_2_-Area Under the Curve, AUC) of hypo/hyperoxemia on mortality and poor neurological outcome at 6-months; 4) the effect of PaO_2_ on outcome according to randomization in the normothermia versus hypothermia group.

### Inclusion and exclusion criteria

Inclusion criteria of the TTM2 trial were patients 18 years of age or older admitted to the hospital after OHCA of non-traumatic or unknown cause with a return of spontaneous circulation (ROSC) requiring ICU admission and mechanical ventilation. Exclusion criteria were the following: unwitnessed OHCA with an initial rhythm of asystole, an interval from ROSC to screening over 180 min, temperature on admission < 30 °C, obvious or suspected pregnancy, intracranial bleeding at admission [22, 23]. For this sub-analysis, we further excluded patients who had no available data on PaO_2_ in the first 24 h from hospital admission.

### Data management and collection

Details on the study procedure and patients’ clinical management have been previously described [22, 23]. Ventilatory management was performed according to local practice. Patients’ data were collected at hospital admission, during the intensive care unit (ICU)-stay, at ICU-discharge, at hospital-discharge, and at 6-month follow-up [22, 23]. Data collected included patients’ demographic characteristics, pre-cardiac arrest comorbidities (including Charlson comorbidity index [26]), location, timing, type and management of cardiac arrest, clinical presentation (presence of shock, ST-elevation myocardial infarction—STEMI), data on ventilator settings/parameters (tidal volume—V_T_, positive end-expiratory pressure—PEEP, respiratory rate—RR, fraction of inspired oxygen—FiO_2_, plateau pressure—Pplat, peak pressure—Ppeak, compliance of respiratory system—Crs), and arterial blood gases (ABG) values (pHa, PaO_2_, partial pressure of carbon dioxide—PaCO_2,_ base excess) and clinical outcomes. Ventilatory settings and ABG values were collected from randomization every 4 h for the first 32 h, and then, every 8 h until day 3 (72 h).

### Clinical outcome measures

Clinical outcome measures were mortality and patients’ neurological outcome at 6-month follow-up, the latter evaluated through the Modified Rankin Scale (mRS). The mRS score for neurologic disability is a 7 categories scoring system, ranging from no symptoms (score 0) to patient’s death (score 6), where poor neurological outcome is defined as a score ranging from 4 to 6. Follow-up data were obtained by study participants through telephone interview, postal questionnaire, or a face-to-face visit. Responses were obtained from patients or from a next of kin in cases of impaired cognitive capacity, which could prevent patient interview.

### Statistical analysis

At baseline, data on patient characteristics, ventilator settings, and ABG were presented as means ± standard deviation, or medians [interquartile range (IQR)] for continuous variables, or as percentages for the categorical ones. The comparisons of means, medians, and frequencies among the three categories for PaO_2_ were carried out using one-way ANOVA, Kruskal–Wallis' test, and chi-square test, respectively. When building a regression model, the process of variable selection comprised an initial model with: (1) patients’ clinical characteristics (age, sex, body mass index—BMI, height, Charlson comorbidity index, state of shock at admission, and STEMI diagnosis on admission); (2) onsite-related cardiopulmonary resuscitation (CPR) related variables (ROSC time, bystander CPR, OHCA physical location, initial cardiac rhythm, witnessed OHCA); (3) treatment variables from the original trial (randomization arm and tympanic temperature at admission); and (4) ABG values and (5) ventilatory settings parameters. From this initial set of covariates, a more parsimonious model was developed by backward elimination using a multivariable fractional polynomial (FP) procedure [27]. The linearity assumption of continuous variables was tested, and the variable transformed with the appropriate FP when the assumption was not met. Risk estimates from the Cox regression and logistic regression models were expressed as hazard ratios (HRs) and Odds ratios (ORs) with 95% confidence intervals (95% CI), respectively. If PaO_2_/PaO_2_-AUC (as continuous) were modeled with a FP, their association with the endpoint was instead depicted through a graph where the HR/OR on the y-scale is plotted against the continuum of the marker.

The independent association between baseline PaO_2_ (or PaO_2_ groups-PaO_2__class) with 6-months mortality was evaluated with Cox regression analysis. As a sensitivity analysis, the area under the receiving operator curve (ROC) curve of all PaO_2_ values (PaO_2_-AUC) was calculated for each patient and tested in a Cox regression for mortality, which was built considering the repeated measures of PaO_2_ as a single time point representing the numerical integration of PaO_2_ values and the time between measurements. Therefore, PaO_2_-AUC represents a sequential (cumulative) integration over time of all PaO_2_ preceding values obtained during the first 72 h since ICU admission. Because we were interested exploring the prognostic value of PaO_2_-AUC on hypoxemia and hyperoxemia, an interaction with PaO_2__class was included in the Cox regression model.

A 2-sided *p* value of < 0.05 was the threshold used for significance in all analyses. Stata 16.1 was used for data clean-up, preparation, and statistical analysis. Further details on statistical methods are presented in the Additional file 1.

## Results

### Characteristics of the patients in the whole population

From a total of 1861 patients included in the TTM2 trial, 443 patients were excluded due to missing values in PaO_2_ in the first 24 h, leaving a sample of 1418 patients (Table 1, Additional file 1: Tables S2, S3). The median age was 65 [IQR = 55–74] years, and 292 (20.6%) were female. At 6-month follow-up, 696 (49.1%) patients were dead, and 740 (55.9%) had poor neurological outcome. Additional file 1: Table S2 and S3 present patients’ clinical characteristics, outcome measures, and ventilator settings, respectively, and according to the different classes of PaO_2_.

**Table 1:** Baseline patients’ characteristics, comorbidities, pre-hospital settings/interventions of the overall population and stratified according to oxygen values

	Overall (*n* = 1418, 100.0%)	PaO_2_ < 60 mmHg (*n* = 79, 5.6%)	PaO_2_ 60–300 mmHg (*n* = 1239, 87.4%)	PaO_2_ > 300 mmHg (*n* = 100, 7.1%)	*p* value
*Baseline patient characteristics*					
Age, years, median (IQR)	65 (55; 74)	66 (57; 75)	65 (55; 74)	66 (56; 74)	0.415
Gender, female, *n* (%)	292 (20.6)	13 (16.5)	250 (20.2)	29 (29.0)	0.071
Height, cm, median (IQR)	175 (170; 180)	176 (170; 180)	175 (170; 180)	170 (165; 179)	0.005
Weight, kg, median (IQR)	80 (73; 90)	85 (80; 93)	80 (73; 91)	80 (70; 88)	0.001
BMI, kg/m^2^, median (IQR)	26.3 (24.1; 29.7)	27.4 (25.0; 30.6)	26.3 (24.1; 29.7)	26.1 (23.3; 28.4)	0.021
*Chronic comorbidities*					
Hypertension, yes, *n* (%)	504 (35.5)	25 (31.6)	449 (36.2)	30 (30.0)	0.115
Diabetes mellitus, yes, *n* (%)	266 (18.8)	15 (19.0)	234 (18.9)	17 (17.0)	0.896
Myocardial infarction, yes, *n* (%)	230 (16.2)	14 (17.7)	201 (16.2)	15 (15.0)	0.244
Percutaneous coronary intervention, yes, *n* (%)	210 (14.8)	14 (17.7)	181 (14.6)	15 (15.0)	0.130
Coronary artery bypass graft, yes, *n* (%)	112 (7.9)	7 (8.9)	98 (7.9)	7 (7.0)	0.219
Heart failure, yes, *n* (%)	145 (10.2)	9 (11.4)	127 (10.3)	9 (9.0)	0.206
Charlson comorbidity index, points, median (IQR)	4.0 (2.0; 5.0)	4.0 (3.0; 6.0)	4.0 (2.0; 5.0)	4.0 (2.3; 5.8)	0.517
*Pre-hospital setting/interventions*					
Location of cardiac arrest, *n* (%)					
Home	741 (52.3)	42 (53.2)	638 (51.5)	61 (61.0)	
Public place	509 (35.9)	27 (34.2)	452 (36.5)	30 (30.0)	
Other	168 (11.8)	10 (12.7)	149 (12.0)	9 (9.0)	0.474
Witnessed cardiac arrest, yes, *n* (%)	1295 (91.3)	71 (89.9)	1131 (91.3)	93 (93.0)	0.753
Bystander performed CPR, yes, *n* (%)	1148 (81.0)	63 (79.7)	1007 (81.3)	78 (78.0)	0.696
Type of rhythm, *n* (%)					
Not shockable	390 (27.5)	24 (30.4)	331 (26.7)	35 (35.0)	
Shockable	1028 (72.5)	55 (69.6)	908 (73.3)	65 (65.0)	0.558
Time of ROSC, minutes, median (IQR)	25 (17; 39)	25 (19; 39)	25 (17; 39)	25(18.3; 35.8)	0.558
TTM randomization treatment, *n* (%)					
Normothermia	712 (50.2)	46 (58.2)	615 (49.6)	51 (51.0)	0.330
Hypothermia	706 (49.8)	33 (41.8)	624 (50.4)	49 (49.0)	

Data are reported as median (interquartile range, IQR) and number (percentage, %). Legend: *n* = number of patients, BMI, body mass index, IBW, ideal body weight, ROSC, return of spontaneous circulation, CPR, cardio-pulmonary resuscitation, TTM, target temperature management

### PaO_2_ distribution and the occurrence of episodes of hypo/hyperoxemia

**Fig. 1:**
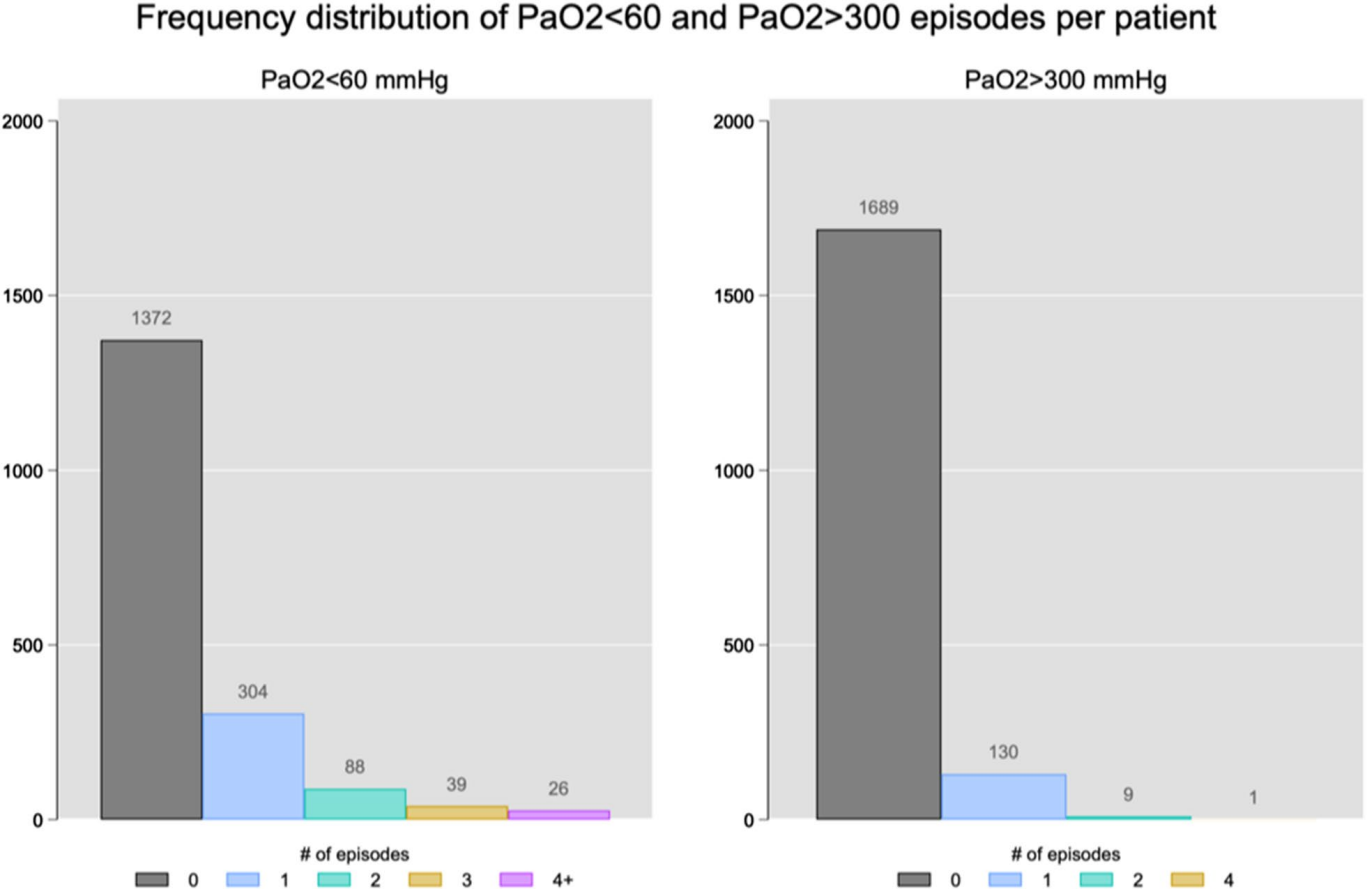
Frequency distribution of arterial partial pressure of oxygen (PaO_2_) classes (conventional thresholds). Number of hypoxemia (PaO_2_ < 60 mmHg) or severe hyperoxemia (PaO_2_ > 300 mmHg) episodes per patient during the first 72 h after intensive care unit admission. This figure was based on all patients included in the cohort, with a percent distribution as follow: Episodes of Hypoxemia = 0 (*n* = 1372 (75.01%), 1 (*n* = 304 (16.62%), 2 (*n* = 88 (4.81%), 3 (*n* = 39 (2.13%), 4 + (*n* = 26 (1.42%). Episodes of Hyperoxemia = 0 (*n* = 1689 (92.35%), 1 (*n* = 130 (7.11%), 2 (*n* = 9 (0.49%), 4 (*n* = 1 (0.05%)

At admission, the median PaO_2_ value in the overall population was 108 mmHg [IQR = 83–163]. Seventy-nine patients (5.6%) presented a PaO_2_ < 60 mmHg (median PaO_2_ = 51 mmHg [IQR = 39.7–56.2]); 100 (7.1%) patients a PaO_2_ > 300 mmHg (median PaO_2_ = 363 mmHg [IQR = 330–433]); and 1239 (87.4%) patients had a PaO_2_ between 60 and 300 mmHg (median PaO_2_ = 108 mmHg [IQR = 85.5–148.5]). PaO_2_ trajectories over the 72 h study period are shown in Additional file 1: Figure S1. Over the study period, 24.9% of patients had at least one episode of PaO_2_ < 60 mmHg and 7.6% of patients had at least one episode of PaO_2_ > 300 mmHg, Fig. 1. In most cases, patients had 1 or 2 episodes over the first 72 h, whereas more than 2 episodes were less frequent. The incidence rates (number of episodes per person in the 72 h follow-up) for PaO_2_ < 60 mmHg and PaO_2_ > 300 mmHg were 0.42 (95% CI 0.39–0.45) and 0.08 (95% CI 0.07–0.10), respectively.

### The association between hypo- and hyperoxemia with 6-months mortality

**Fig. 2:**
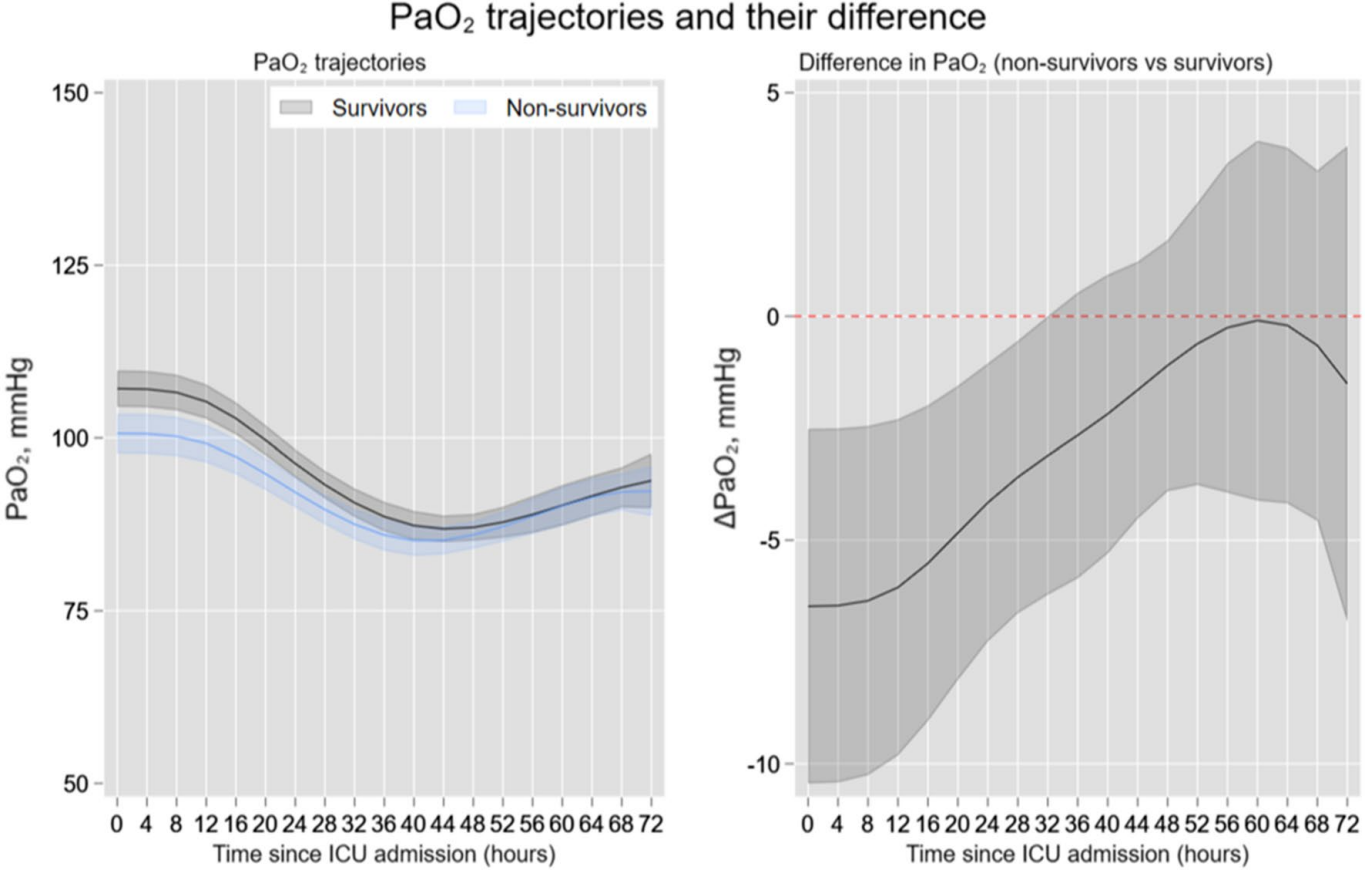
Adjusted hourly trajectories of partial pressure of oxygen according to 6-month survival status. Left panel shows the predicted partial pressure of oxygen (PaO_2_) trajectories according to survival status. Right panel shows the PaO_2_ differences between survivors and non-survivors at each time point. For this analysis, mixed regression model included a random intercept on patients ID and a random coefficient on the time variable (time elapsed between measurements). These predicted trajectories were adjusted for TTM2 randomization arms, age (year), gender, Charlson comorbidity index, state of shock at admission, return to spontaneous circulation-ROSC- time, initial cardiac rhythm (shockable vs non-shockable), witnesses of cardiac arrest, respiratory rate (breath/min), plateau pressure (cmH_2_O),positive end expiratory pressure (cmH_2_O), arterial partial pressure of carbon dioxide, PaCO_2_ (mmHg), pH, Base excess (mEq/L), and fraction of inspired O_2_ (%). Right panel confirmed that the differences between these two trajectories (survivors/non-survivors) are statistically significant up to the first 32 h of measurement (omnibus *p* value = 0.0074). ICU, Intensive Care Unit

**Fig. 3:**
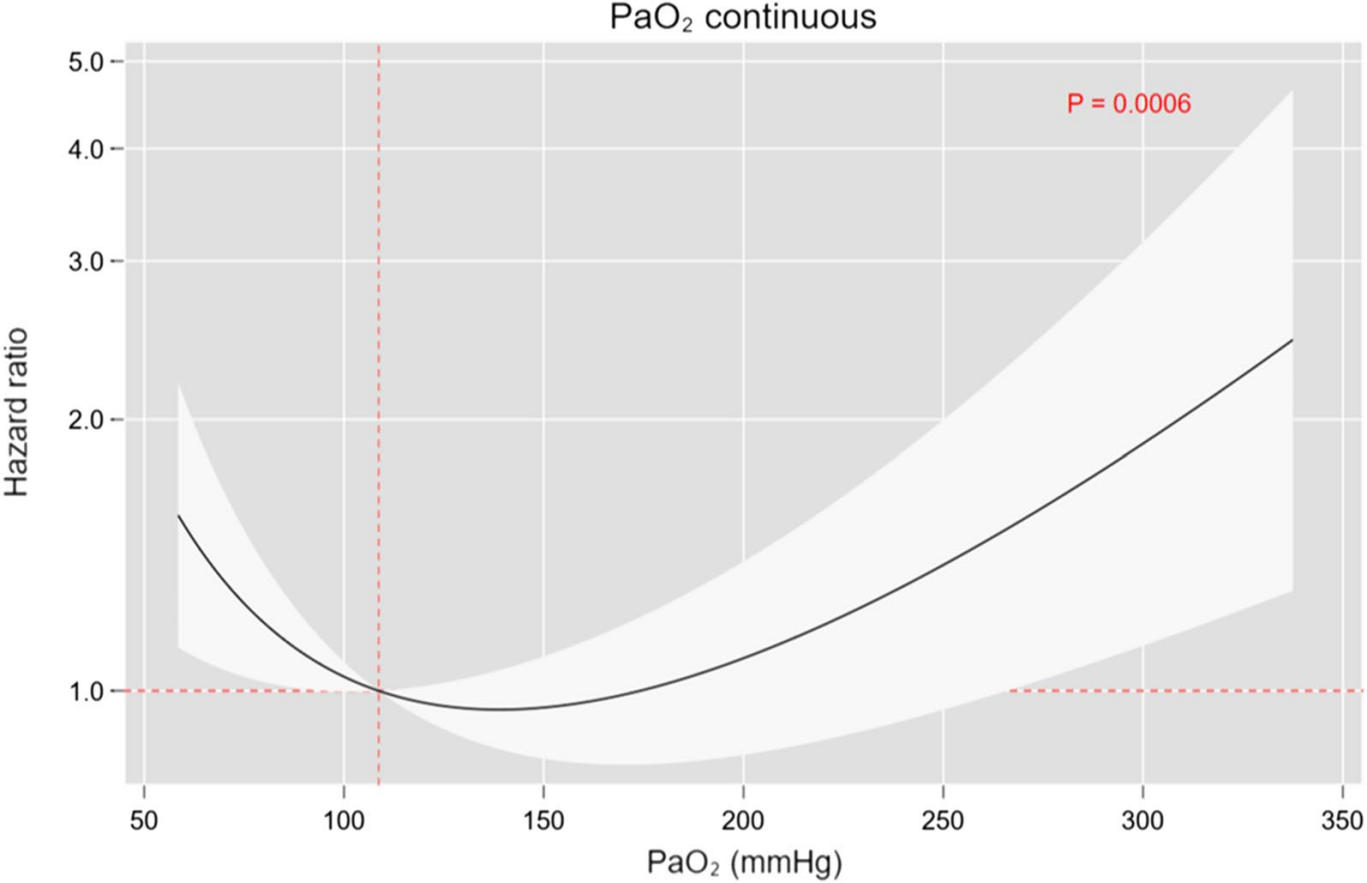
Arterial partial pressure of oxygen (PaO_2_) mortality risk profile. In this Cox regression, PaO_2_ was modeled with a fractional polynomial (FP) of second degree FP [0–1], and included the following covariates: TTM2 randomization group, tympanic temperature at admission, age (years), gender, Charlson comorbidity index, cardiac arrest witnessed, time to return to spontaneous circulation, ROSC (min), bystander performed cardiopulmonary resuscitation, CPR, shockable rhythm, cardiac arrest location (home, public place, other), shock diagnosis on admission, ST-Elevated myocardial infarction (STEMI) diagnosis on admission, respiratory rate (breath/min), positive end-expiratory pressure, arterial partial pressure of carbon dioxide (PaCO_2_) (mmHg), pHa, and Base excess (mEq/L), Driving pressure (cmH_2_0), and mechanical power (J/min). Along the PaO_2_ continuum, values before and after its median (108.7 mmHg and used as reference—see vertical line in red) were statistically associated with mortality if the 95% confidence interval (CI) did not cover the y-line of 1 (horizontal line in red)

Figure 2 presents the adjusted PaO_2_ trajectories according to survival status. PaO_2_ values decreased significantly until the 40^th^ hour and then, leveled-off afterward both in survivors and non-survivors. The differences between the two trajectories according to survival status were statistically significant up to the first 32 h of measurement (omnibus *p* value = 0.007). Higher PaO_2_ values were associated with better survival. The Kaplan–Meier curve (Additional file 1: Figure S2) suggested a trend toward better survival in the normoxemia group, compared to both the hypoxemia and severe hyperoxemia groups, although not statistically significant. At multivariable Cox regression, PaO_2_ followed a U-shape risk profile, demonstrating that both hypo- and hyperoxemia were independently associated with higher mortality rates (omnibus *p* value = 0.0006; Fig. 3).

### Definition of the “best” threshold of hypoxemia and hyperoxemia associated with 6-months mortality

**Fig. 4:**
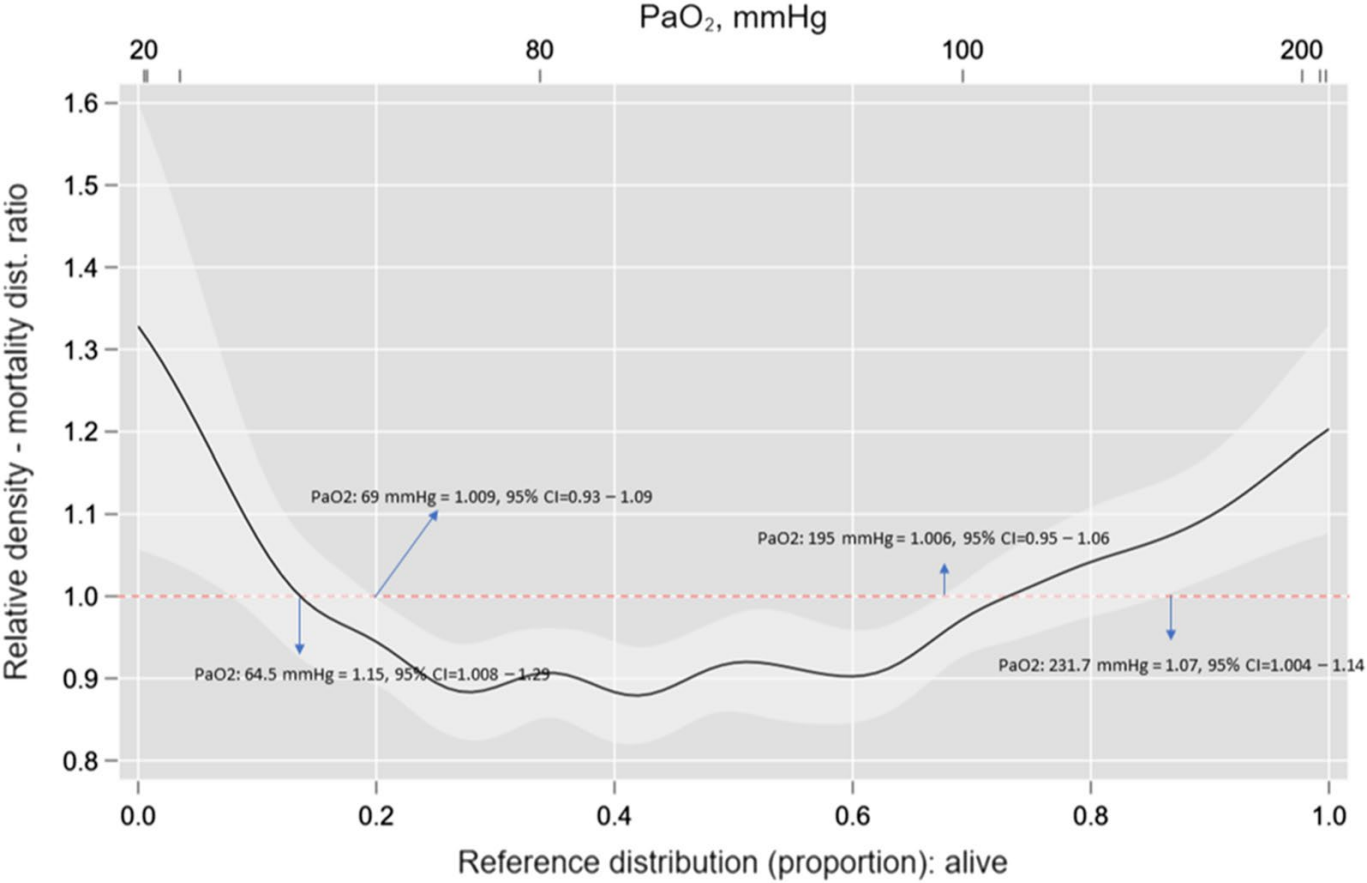
Relative distribution analysis for the definition of the best cut-off of arterial partial pressure of oxygen (PaO_2_) associated with mortality. Best cutoff point along the continuum of the marker that separated survivors versus non-survivors at the end of the follow-up. In this analysis, the quantile (or proportion) distribution of the marker survivors (plotted on the *x*-axis plus the corresponding marker values at the top) is plotted against the proportion ratio of the marker distribution for non-survivors

**Fig. 5:**
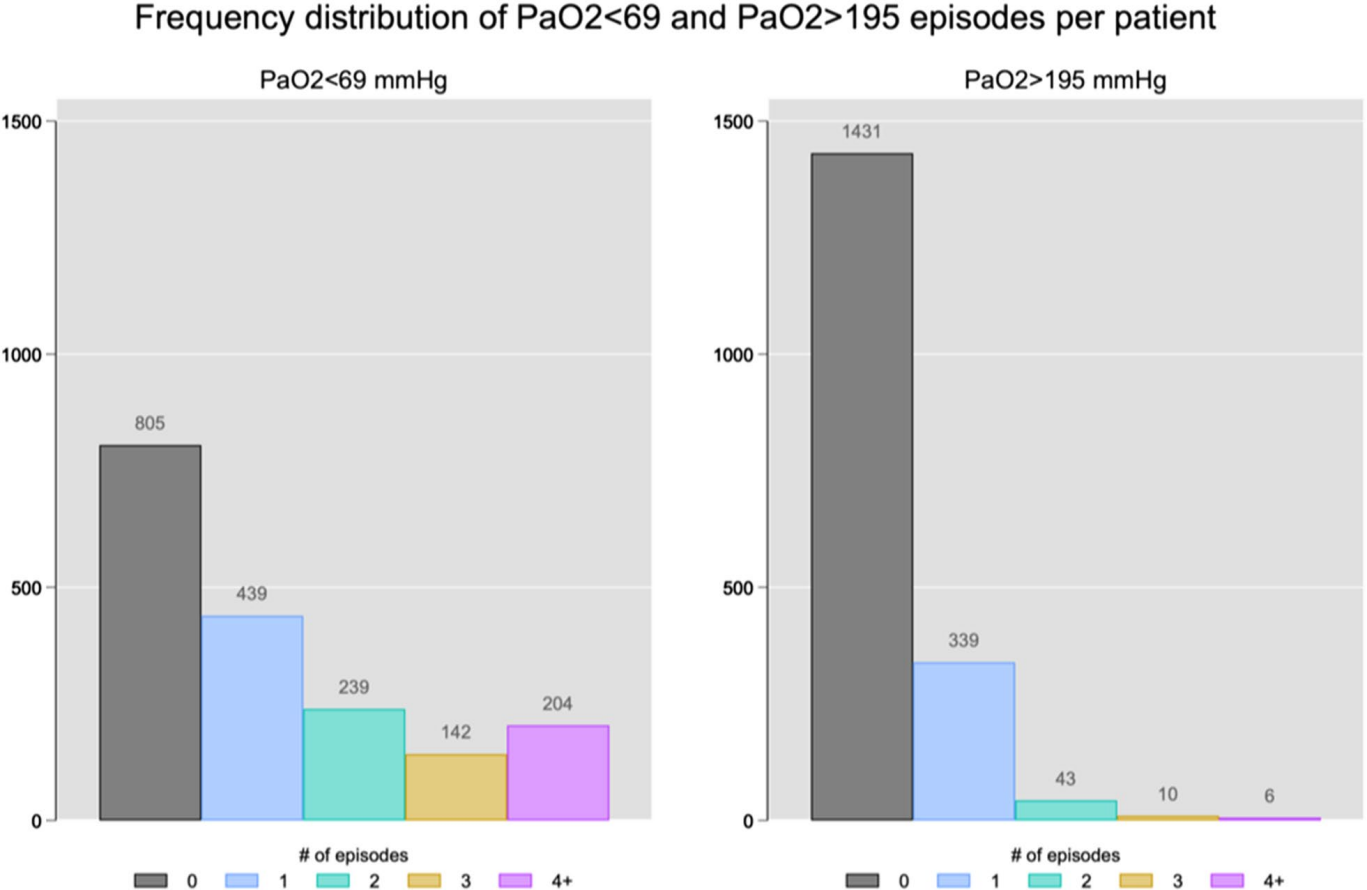
Frequency distribution of arterial partial pressure of oxygen (PaO_2_) classes (according to best threshold). Numbers of hypoxemia/hyperoxemia episodes per patient during the first 72 of mechanical ventilation. This figure was based on all patients included in the cohort, with a percent distribution as follow: Episodes of Hypoxemia = 0 (*n* = 805 (44.01%), 1 (*n* = 439 (24.00%), 2 (*n* = 239 (13.07%), 3 (*n* = 142 (7.76%), 4 + (*n* = 204 (11.05%). Episodes of Hyperoxemia = 0 (*n* = 1431 (76.90%), 1 (*n* = 339 (18.53%), 2 (*n* = 43 (2.35%), 3 (*n* = 10 (0.55%), 4 + (*n* = 6 (0.33%)

Figure 4 shows the “best” threshold of hypoxemia and hyperoxemia for the prediction of 6-month mortality in our cohort. The best cut-off point for hypoxemia was a PaO_2_ of 69 mmHg (Risk Ratio = 1.009, 95% CI 0.93–1.09) and for hyperoxemia was a PaO_2_ of 195 mmHg (Risk Ratio = 1.006, 95% CI 0.95–1.06). The characteristics of the patients according to the best thresholds calculated are shown in Additional file 1: Table S4–S6. At admission, 165 patients (11.6%) presented with hypoxemia (median value 60 mmHg [IQR = 51.7–65.2]), 263 (18.5%) with hyperoxemia (273 mmHg [IQR = 231.7–342.7]), and 990 patients (69.8%) with normoxemia (105 mmHg [IQR = 87–133]). Over the study period, 55.9% of patients had at least one episode of hypoxemia and 21.7% had at least one episode of hyperoxemia, and in most cases patients experienced only 1 or 2 episodes of hypoxemia and/or hyperoxemia over the first 72 h of mechanical ventilation. The incidence (number episodes per person in the 72 h follow-up) for hypoxemia and hyperoxemia considering the best thresholds was 1.34 (95% CI 1.29–1.40) and 0.26 (95% CI 0.24–0.29), respectively (Fig. 5).

### Dose of oxygen and interaction between oxygen values and TTM2-arms.

Additional file 1: Figure S3 shows the hypoxemia and hyperoxemia mortality risk difference considering the exposure over time or “dose” of oxygen defined as PaO_2_-AUC. PaO_2_-AUC for hyperoxemia showed to be associated with higher mortality risk as compared to normoxemia (interaction *p* value = 0.0039).

Additional file 1: Figure S4 shows the interaction between PaO_2_ and TTM2-arms (hypothermia versus normothermia). No difference was observed on the effect of PaO_2_ on mortality between the TTM2-randomization groups (interaction *p* value = 0.997). HRs of hypoxemia and hyperoxemia on mortality for the hypothermia group were 1.07 (95% CI 0.58–1.98; *p* = 0.82), and 1.38 (95% CI 0.82–2.32; *p* = 0.22), respectively.

### The association between hypo- and hyperoxemia with neurological outcome

No differences were observed in the trajectories of PaO_2_ values in the first 72 h according to poor and good neurological status (omnibus value *p* = 0.35). Also, the distribution of the mRS score was not different among PaO_2_ classes (Additional file 1: Figure S5, *p* = 0.55). At multivariate analysis, no significative association with poor neurological outcome (mRS = 4–6) was observed (omnibus value, *p* = 0.63), even considering separately mRS 4 and 5 (Additional file 1: Figure S6). Accordingly, we were not able to find a best cut-off point for neurological outcome (Additional file 1: Figure S7).

## Discussion

In a large cohort of OHCA patients included in an international multicenter randomized clinical trial, we found that: (1) hypoxemia and severe hyperoxemia events are uncommon after OHCA, considering the conventional thresholds suggested in the literature; (2) the “best” cutoff values of oxygen associated with the risk for mortality were a PaO_2_ below 69 mmHg and above 195 mmHg; with the use of these cut-offs, the incidence of episodes of hypoxemia and hyperoxemia markedly increased; (3) hypoxemia and hyperoxemia are independently associated with 6-months mortality but not with neurological outcome; the time-exposure (or “dose”) of hyperoxemia was associated with 6-months mortality; and 4) these results were consistent across the group of randomization (normothermia or hypothermia).

To the best of our knowledge, this is the largest prospective study exploring the targets of oxygen as well as the association of hypoxemia and hyperoxemia with outcome in a homogeneous population of OHCA patients. We believe that our results are relevant and confirm not only the important effects of hypoxemia but also of hyperoxemia on 6-months mortality. In addition, we identified new thresholds of PaO_2_ which are at risk for poor outcome.

Several studies highlighted the importance of maintaining appropriate ventilation targets and levels of PaO_2_ in OHCA patients [27]. Post-cardiac arrest syndrome includes a number of pathophysiological mechanisms such as brain edema, reperfusion injury and oxidative stress, which can lead to neuronal damage and brain injury [28]. Hypoxemia caused by cardiac arrest yields to an alteration of cerebral metabolism, neuronal cell injury and death [7, 29].

The occurrence of hypoxemia and hyperoxemia is variable in the literature, with overall incidence of about 19% for hypoxemia [7] and between 3 and 60% for hyperoxemia [7, 25, 30]. Considering the conventional thresholds, the incidences of episodes of hypoxemia and severe hyperoxemia in our study were compatible with previous literature. The use of new “best” thresholds for oxygenation compared to traditional ones led to a marked increase in the number of patients exposed to at least one episode of hypoxemia or hyperoxemia.

The PaO_2_ threshold responsible for the onset of hypoxic neuronal damage is not completely defined, and it is generally considered at 60 mmHg [31–34]. This value could underestimate the risk of hypoxemia in the OHCA population as the “best” lower threshold associated with increased mortality was found at PaO_2_ of 69 mmHg.

Although recommendations suggest to give the maximum feasible inspired oxygen during CPR [8, 18, 35] to avoid hypoxemia [36, 37], recent evidence suggests a possible harmful effect also of hyperoxemia after OHCA [38]. A systematic review reported higher mortality in hyperoxemic compared to normoxemic patients with cardiac arrest and extracorporeal life support, but not in other groups of patients [39]. Another recent meta-analysis of observational studies [40] showed that severe hyperoxemia (PaO_2_ > 300 mmHg) was associated with worse outcome, especially if hyperoxemia occurred during the first 36 h after cardiac arrest. However, high heterogeneity was found among the studies included in the meta-analysis, regarding the threshold of oxygen adopted, patient selection, the use of TTM, outcome measurement, methods of analyzing blood gas and often lack a pre-defined sampling protocol [20, 21, 41]. Many studies just considered PaO_2_ values in the very early phases from ROSC [42], did not evaluate the duration of hyperoxemia (the dose), had limited sample sizes, or had retrospective designs or prospective design with a post hoc analysis [7, 8, 18, 43]. In the present preplanned study, both hypoxemia and hyperoxemia as well as the dose (AUC) of hyperoxemia over time were associated with mortality. This implies that the pathophysiological effect of hyperoxemia importantly depends not only on the intensity, but also on the duration of the exposure to high oxygen values. Also, the best upper threshold of PaO_2_ associated with the risk for mortality was above 195 mmHg. This point is of critical importance and makes our results unique, potentially explaining why in previous studies using the conventional threshold of 300 mmHg a non-consistent association with outcome was found [41, 44–46]. We hypothesize that the risk for hyperoxemia might have been underestimated considering the traditional thresholds, and that in the post-ROSC phase clinicians should pay attention in the titration of oxygen to lower levels than thought before.

Different oxygen targets have been proposed by trials on oxygen [47–49], and the recent BOX trial [49], which compared 2 targets of PaO_2_ 68–75 mmHg vs 98–105 mmHg, showed similar incidence of death or severe disability or coma among groups, suggesting that question remains especially about the higher target of oxygen to be applied in this population, which requires further investigation.

When splitting the patients according to the use of hypothermia and normothermia, no statistically differences were found in outcome between the two groups, thus suggesting that temperature 33 °C does not improve oxygen tolerance and could further explain the lack of protective effect of hypothermia [23]. The fact that hypoxemia and hyperoxemia were not associated with poor neurological outcome (mRS 4 and 5) might be explained by different factors. Firstly, the scale used to evaluate neurological disability does not specifically account for specific cognitive dysfunction; further, neurological outcome may be affected by many different post-acute factors such as systemic complications or secondary brain damage during the ICU stay, in the hospital, or during rehabilitation. In particular, despite we used a robust statistical model which took in consideration several confounding factors, oxygen derangements might be a marker for systemic clinical events that can lead to an increase in mortality, i.e., pneumonia, sepsis, without a definitive effect on neurological outcome.

### Limitations

This study has several limitations. Firstly, although this was a preplanned secondary analysis of the TTM2 trial, this is an observational study, and our results should be regarded as hypothesis-generating, and we cannot make any causality statements from our results. A randomized clinical trial will be in fact necessary to confirm our findings, with the aim to explore the effect of oxygen more deeply on neurological outcome and the interaction of oxygen derangements with systemic factors.

Secondly, we hypothesized that oxygen pressure in-between PaO_2_ measurement was linear, and we were not able to account for short-term variations of PaO_2_. Nevertheless, the present study includes the highest number of available data on PaO_2_ measures with serial measurements. Third, although this was a preplanned study, some information is lacking in eCRF, and some data are missing in the database. Finally, the conventional thresholds used in this analysis were adopted according to robust observational studies, but these values present important heterogeneity in the literature [18, 25, 47], with no definitive conclusions regarding the optimal oxygen targets, especially for the higher threshold of oxygen. The ongoing Mega-ROX trial [48] is exploring two different levels of oxygen mainly based on SpO_2_ and a recently published RCT [49], compared 2 targets of PaO_2_ with higher target of 98–105 mmHg. Our results can pave the way to the definition of further RCTs and better define the best thresholds of oxygenation to be applied in this population.

## Conclusions

In mechanically ventilated patients after out of hospital cardiac arrest, we found novel “best” cutoff values of oxygen associated with the risk for mortality at PaO_2_ below 69 mmHg and above 195 mmHg; with the use of these cut-offs, episodes of hypoxemia and hyperoxemia are common in this population. Both hypoxemia and hyperoxemia are associated with higher 6-months mortality, and this may be mediated by the timing exposure to high values of oxygen. More cautious titration of oxygen levels should be considered in this group of patients until stronger evidence is available.

## Supplementary Information

**Additional file 1:** Additional statistical analysis of subgroups population and association with outcome.

## Data Availability

The datasets generated and/or analyzed during the current study are not publicly available but are available from the corresponding author on reasonable request.

## Abbreviations

ABG: Arterial blood gas
AUC: Area under curve
BMI: Body mass index
CI: Confidence interval
CO_2_: Carbon dioxide
Crs: Respiratory system compliance
eCRF: Electronic case record form
CPR: Cardiopulmonary resuscitation
FiO_2_: Fraction of inspired oxygen
FP: Fractional polynomial
HR: Hazard ratio
ICU: Intensive care unit
IQR: Interquartile range
LOS: Length of stay
mRS: Modified Rankin Scale
OHCA: Out of hospital cardiac arrest
OR: Odds ratio
PaCO_2_: Arterial partial pressure of CO_2_
PaO_2_: Arterial partial pressure of oxygen
PEEP: Positive end-expiratory pressure
pHa: Arterial pH
PI: Principal investigator
Ppeak: Peak pressure
Pplat: Plateau pressure
ROSC: Return of spontaneous circulation
RR: Respiratory rate
STEMI: ST elevation myocardial infarction
STROBE: STrengthening the Reporting of OBservational studies in Epidemiology
TTM2: Target temperature management 2 Trial
V_T_: Tidal volume
