# Supplementary material for: Oxygen targets and 6-month outcome after out of hospital cardiac arrest: a pre-planned sub-analysis of the targeted hypothermia versus targeted normothermia after Out-of-Hospital Cardiac Arrest (TTM2) trial
Source: Crit Care. 2022 Oct 21;26:323. doi: 10.1186/s13054-022-04186-8 (PMC9585831; doi:10.1186/s13054-022-04186-8)
Supplement: Supplementary file 1 — Additional file 1: Additional statistical analysis of subgroups population and association with outcome. [file 13054_2022_4186_MOESM1_ESM.docx]

**Electronic supplementary material (ESM)**

Oxygen and 6 months outcome after out of hospital cardiac arrest: a preplanned sub-analysis of the Targeted Hypothermia versus Targeted Normothermia after Out-of-Hospital Cardiac Arrest (TTM2) trial

Chiara Robba, Rafael Badenes, Denise Battaglini, Lorenzo Ball, Iole Brunetti, Filippo Sanfilippo, Janus C Jakobsen, Gisela Lilja, Hans Friberg, Pedro D Wendel-Garcia, Paul J Young, Glenn Eastwood, Michelle S Chew, Johan Unden, Matthew Thomas, Michael Joannidis, Alistair Nichol, Andreas Lundin, Jacob Hollenberg, Naomi Hammond, Manoj Saxena, Annborn Martin, Miroslav Solar, Fabio Silvio Taccone, Josef Dankiewicz, Niklas Nielsen, Anders Grejs, Florian Ebner and Paolo Pelosi

**Table of content**

Table S1. Strengthening the Reporting of Observational Studies in Epidemiology (STROBE)” statement guidelines [page 3-5].

Table S2. Patients’ clinical outcomes in the overall population and stratified according to oxygen values according to conventional thresholds [page 6].

Table S3. Ventilatory settings, respiratory mechanics and gas exchange at admission stratified according to oxygen values according to conventional thresholds [page 7].

Table S4. Characteristics of the patients, in the overall population and stratified according to the best threshold of oxygen values for mortality [page 8-9].

Table S5. Patients’ clinical outcomes in the overall population and stratified according to the new oxygen thresholds [page 10].

Table S6. Ventilatory setting, respiratory mechanics and gas exchange at ICU admission stratified according to the new oxygen thresholds [page 11].

Figure S1. Hourly oxygen trajectories in the overall population, and by survival status (survivors and non survivors) [page 12].

Figure S2 a, b. Kaplan-Meier curve considering conventional and new thresholds. Most deaths occurred during the first 30 days of the follow-up [page 13-14].

Figure S3. Hypoxemia and hyperoxemia mortality risk difference considering the dose of oxygen (exposure over time, area under the curve, PaO_2_-AUC) [page 15].

Figure S4. Interaction between PaO_2_ (continuous) and TTM2-arms [page 16].

Figure S5. Frequency distribution of modified Rankin Score (mRS) among partial pressure of oxygen, PaO_2_ classes [page 17].

Figure S6. Analysis for the definition of the best cut off point of oxygen values for the prediction of poor neurological outcome [page 18].

Figure S7. Predicted probability of achieving a modified Rankin Score (mRS) 4 or 5 according to PaO_2_ values [page 19].

*Further details on statistical analysis methods*

Using ordinal logistic regression, we estimated the probability that specific value of PaO_2_ (or PaO_2__class) was associated with poor neurological outcome. Using relative distribution analysis, we searched for the best cut-point along the continuum of the PaO_2_ that separated 6-months survivors from non-survivors. To account for interdependence among repeated measures, the models included a cluster-based adjustment of the standard error estimation. Linear mixed regression was used to compare the longitudinal trajectories of hourly measured PaO_2_ among survival status. For this longitudinal analysis, a repeated measure dataset (included hourly ventilatory markers collected every 4 hours during the first 72 hours after ICU admission) was used and included 18716 observations, after excluding missing values on survival status and PaO_2_. A similar set of covariates as in the Cox model, were used for building the adjusted model. To account for the longitudinal nature of the data (interdependence among repeated measures), the model included a random effect (intercept) on patient ID and a random coefficient on the time elicited between PaO_2_ measures. CR and RB had full access to all the data in the study and takes responsibility for its integrity and the data analysis.

**Table S1. “Strengthening the Reporting of Observational Studies in Epidemiology (STROBE)” statement guidelines.**

|  | **Item No.** | **Recommendation** | **Page  No.** | **Relevant text from manuscript** |
| --- | --- | --- | --- | --- |
| **Title and abstract** | 1 | (*a*) Indicate the study’s design with a commonly used term in the title or the abstract | 1 |  |
|  |  | (*b*) Provide in the abstract an informative and balanced summary of what was done and what was found | 4 |  |
| **Introduction** | | | |  |
| Background/rationale | 2 | Explain the scientific background and rationale for the investigation being reported | 8 |  |
| Objectives | 3 | State specific objectives, including any prespecified hypotheses | 9-10 |  |
| **Methods** | | | |  |
| Study design | 4 | Present key elements of study design early in the paper | 10-12 |  |
| Setting | 5 | Describe the setting, locations, and relevant dates, including periods of recruitment, exposure, follow-up, and data collection | 10-12 |  |
| Participants | 6 | (*a*) *Cohort study*—Give the eligibility criteria, and the sources and methods of selection of participants. Describe methods of follow-up | 10-12 |  |
|  |  |  |  |  |
| Variables | 7 | Clearly define all outcomes, exposures, predictors, potential confounders, and effect modifiers. Give diagnostic criteria, if applicable | 9-10 |  |
| Data sources/ measurement | 8* | For each variable of interest, give sources of data and details of methods of assessment (measurement). Describe comparability of assessment methods if there is more than one group | 9-12 |  |
| Bias | 9 | Describe any efforts to address potential sources of bias | 9-12 |  |
| Study size | 10 | Explain how the study size was arrived at | 9-12 |  |

| Quantitative variables | 11 | Explain how quantitative variables were handled in the analyses. If applicable, describe which groupings were chosen and why | 12-13 |  |
| --- | --- | --- | --- | --- |
| Statistical methods | 12 | (*a*) Describe all statistical methods, including those used to control for confounding | 12-13 |  |
|  |  | (*b*) Describe any methods used to examine subgroups and interactions |  |  |
|  |  | (*c*) Explain how missing data were addressed |  |  |
|  |  |  |  |  |
|  |  | (d) Describe any sensitivity analyses | NA |  |
| **Results** | | | | |
| Participants | 13* | (a) Report numbers of individuals at each stage of study—eg numbers potentially eligible, examined for eligibility, confirmed eligible, included in the study, completing follow-up, and analyzed | 9-12 |  |
|  |  | (b) Give reasons for non-participation at each stage |  |  |
|  |  | (c) Consider use of a flow diagram |  |  |
| Descriptive data | 14* | (a) Give characteristics of study participants (eg demographic, clinical, social) and information on exposures and potential confounders | 9-12 |  |
|  |  | (b) Indicate number of participants with missing data for each variable of interest |  |  |
|  |  | (c) *Cohort study*—Summaries follow-up time (eg, average and total amount) |  |  |
| Outcome data | 15* | *Cohort study*—Report numbers of outcome events or summary measures over time | 9-12 |  |
|  |  | *Case-control study—*Report numbers in each exposure category, or summary measures of exposure |  |  |
|  |  | *Cross-sectional study—*Report numbers of outcome events or summary measures |  |  |
| Main results | 16 | (*a*) Give unadjusted estimates and, if applicable, confounder-adjusted estimates and their precision (eg, 95% confidence interval). Make clear which confounders were adjusted for and why they were included | 14-18 |  |
|  |  | (*b*) Report category boundaries when continuous variables were categorized |  |  |
|  |  | (*c*) If relevant, consider translating estimates of relative risk into absolute risk for a meaningful time period |  |  |
| Other analyses | 17 | Report other analyses done—eg analyses of subgroups and interactions, and sensitivity analyses | ESM |  |
| **Discussion** |  |  |  |  |
| Key results | 18 | Summaries key results with reference to study objectives | 18 |  |
| Limitations | 19 | Discuss limitations of the study, taking into account sources of potential bias or imprecision. Discuss both direction and magnitude of any potential bias | 21 |  |
| Interpretation | 20 | Give a cautious overall interpretation of results considering objectives, limitations, multiplicity of analyses, results from similar studies, and other relevant evidence | 18-21 |  |
| Generalizability | 21 | Discuss the generalizability (external validity) of the study results | 18-21 |  |
| **Other information** |  |  |  |  |
| Funding | 22 | Give the source of funding and the role of the funders for the present study and, if applicable, for the original study on which the present article is based. | 23 |  |

*Give information separately for cases and controls in case-control studies and, if applicable, for exposed and unexposed groups in cohort and cross-sectional studies.

**Note:** An Explanation and Elaboration article discusses each checklist item and gives methodological background and published examples of transparent reporting. The STROBE checklist is best used in conjunction with this article (freely available on the Web sites of PLoS Medicine at http://www.plosmedicine.org/, Annals of Internal Medicine at http://www.annals.org/, and Epidemiology at http://www.epidem.com/). Information on the STROBE Initiative is available at www.strobe-statement.org

**Table S2. Patients’ clinical outcomes in the overall population and stratified according to oxygen values according to conventional thresholds.** Data are expressed as median (interquartile range, IQR). ICU, intensive care unit; STEMI, ST elevation myocardial infarction; LOS, length of stay; mRS, modified Rankin Scale. mRS binary defined as poor outcome if points 4-6, good outcome if points 1-3.

|  | **Overall**  **(n=1418, 100.0%)** | **PaO_2_ <60 mmHg**  **(n=79, 5.6%)** | **PaO_2_ 60-300 mmHg**  **(n=1239, 87.4%)** | **PaO_2_ >300 mmHg**  **(n=100, 7.1%)** | **p-**  **value** |
| --- | --- | --- | --- | --- | --- |
| **Neurologic aloutcome** |  |  |  |  |  |
| mRS at 6 months, points, median (IQR) | 6.0 (1.0; 6.0) | 6.0 (2.0; 6.0) | 6.0 (1.0; 6.0) | 6.0 (2.0; 6.0) | 0.180 |
| Poor neurological outcome, n (%) | 756 (54.3) | 46 (59.7) | 650 (53.4) | 60 (60.6) | 0.235 |
| **Hospital / ICU admission** |  |  |  |  |  |
| Shock diagnosis on admission, yes, n (%) | 424 (29.9) | 34 (43.0) | 364 (29.4) | 26 (26.0) | 0.025 |
| STEMI diagnosis on admission, yes, n (%) | 553 (39.0) | 32 (40.5) | 488 (39.4) | 33 (33.0) | 0.003 |
| ICU LOS, days, median (IQR) | 5.1 (3.0; 8.7) | 5.0 (2.7; 8.9) | 5.1 (3.0; 8.7) | 5.0 (3.1; 7.6) | 0.881 |
| Hospital LOS, days, median (IQR) | 9.5 (5.0; 17.0) | 9.6 (3.0; 19.0) | 9.6 (5.0; 17.4) | 8.0 (4.0; 14.9) | 0.197 |
| Time to extubation, days, median (IQR) | 3.6 (1.9; 6.0) | 3.3 (1.9; 6.0) | 3.6 (1.9; 6.1) | 3.7 (2.0; 5.5) | 0.905 |
| Ventilator free days at 30 days, days, median (IQR) | 26.4 (23.8; 28.1) | 26.7 (23.6; 28.1) | 26.4 (23.8; 28.1) | 26.5 (24.4; 28.1) | 0.847 |
| Ventilator free days before ICU discharge, days, median (IQR) | 1.0 (0.0; 2.0) | 0.0 (0.0; 1.5) | 1.0 (0.0; 2.0) | 0.0 (0.0; 1.3) | 0.530 |
| Discharged alive from ICU, n (%) | 893 (63.0) | 48 (60.8) | 789 (63.7) | 56 (56.0) | 0.284 |

**Table S3. Ventilatory settings, respiratory mechanics and gas exchange at admission stratified according to oxygen values according to conventional thresholds.** Data are expressed as median (interquartile range, IQR). Table legend: PBW, predicted body weight; PaO_2_, arterial partial pressure of oxygen; PaCO_2_, arterial partial pressure of carbon dioxide; FiO_2_, fraction of inspired oxygen. Ventilatory ratio was calculated as = (minute ventilation × PaCO_2_) / (PBW × 100 × 37.5).

|  | **Overall**  **(n=1418, 100.0%)** | **PaO_2_ <60 mmHg**  **(n=79, 5.6%)** | **PaO_2_ 60-300 mmHg**  **(n=1239, 87.4%)** | **PaO_2_ >300 mmHg**  **(n=100, 7.1%)** | **p-**  **value** |
| --- | --- | --- | --- | --- | --- |
| **Ventilatory setting and pulmonary mechanics at admission** |  |  |  |  |  |
| Respiratory rate, breaths/min, median (IQR) | 16.0 (14.0; 20.0) | 16.0 (14.0; 18.5) | 16.0 (15.0; 20.0) | 16.0 (14.0; 18.0) | 0.227 |
| Positive end-expiratory pressure, cmH_2_O, median (IQR) | 6.0 (5.0; 8.0) | 7.0 (5.0; 10.0) | 6.0 (5.0; 8.0) | 6.0 (5.0; 8.0) | 0.101 |
| Plateau pressure, cmH_2_O, median (IQR) | 20.0 (17.0; 24.0) | 21.0 (19.0; 26.3) | 20.0 (17.0; 23.0) | 20.0 (15.0; 25.0) | 0.075 |
| Tidal volume, mL, median (IQR) | 500 (445; 560) | 508 (462; 600) | 500 (440; 560) | 500 (450; 600) | 0.245 |
| Tidal volume, mL/kg PBW, median (IQR) | 7.2 (6.4; 8.2) | 7.4 (6.4; 9.3) | 7.1 (6.4; 8.2) | 7.5 (6.7; 8.5) | 0.071 |
| Driving pressure, cmH_2_O, median (IQR) | 13.0 (10.0; 16.0) | 14.5 (12.3; 16.0) | 13.0 (10.0; 16.0) | 12.0 (10.0; 17.0) | 0.265 |
| Compliance of the respiratory system, mL/cmH_2_O, median (IQR) | 40 (31; 50) | 36 (30; 47) | 40 (31; 50) | 41 (29; 59) | 0.368 |
| Mechanical Power, j/min, median (IQR) | 16.2 (12.5; 21.6) | 17.0 (15.0; 23.5) | 16.2 (12.5; 21.5) | 15.3 (12.1; 24.4) | 0.201 |
| Ventilatory ratio, median (IQR) | 1.5 (1.2; 1.8) | 1.6 (0.9; 2.1) | 1.5 (1.2; 1.8) | 1.4 (1.1; 1.9) | 0.854 |
| **Gas exchange** |  |  |  |  |  |
| Fraction of inspired oxygen (FiO_2_), %, median (IQR) | 60 (50; 88) | 70 (50; 100) | 60 (50; 80) | 100 (50; 100) | 0.000 |
| PaO_2_, mmHg, median (IQR) | 108.7 (83.2; 163.0) | 51.0 (39.7; 56.2) | 108.0 (85.5; 148.5) | 363.3 (330.5; 433.6) | 0.000 |
| PaO_2_/ FiO_2_ ratio, mmHg, median (IQR) | 195.0 (122.8; 307.3) | 56.2 (45.7; 83.6) | 192.8 (130.1; 284.9) | 474.7 (373.4; 670.8) | 0.000 |
| PaCO_2_, mmHg, median (IQR) | 45.7 (39.7; 53.2) | 48.8 (37.7; 58.1) | 45.7 (39.8; 53.0) | 42.7 (37.0; 51.7) | 0.020 |
| pHa, median (IQR) | 7.3 (7.2; 7.3) | 7.2 (7.1; 7.3) | 7.3 (7.2; 7.3) | 7.2 (7.2; 7.3) | 0.003 |
| Base excess, mEq/L, median (IQR) | -6.5 (-10.0; -3.7) | -8.0 (-10.5; -4.9) | -6.2 (-9.8; -3.5) | -7.4 (-12.4; -4.3) | 0.005 |

**Table S4. Characteristics of the patients, in the overall population and stratified according to the best threshold of oxygen values for mortality.** Table legend: data are reported as median (interquartile range, IQR), and number (percentage, %). n=number of patients; BMI, body mass index; IBW, ideal body weight; ROSC, return of spontaneous circulation; CPR, cardio-pulmonary resuscitation; TTM, target temperature management.

|  | **Overall**  **(n=1418, 100.0%)** | **PaO_2_<69 mmHg**  **(n=165, 11.6%)** | **PaO_2_ 69-195 mmHg**  **(n=990, 69.8%)** | **PaO_2_ >195 mmHg**  **(n=263, 18.5%)** | **p-**  **value** |
| --- | --- | --- | --- | --- | --- |
| **Baseline patient characteristics** |  |  |  |  |  |
| Age, years, median (IQR) | 65 (55; 74) | 66 (58; 75) | 65 (55; 74) | 65 (55; 74) | 0.291 |
| Gender, female, n (%) | 292 (20.6) | 29 (17.6) | 190 (19.2) | 73 (27.8) | 0.006 |
| Height, cm, median (IQR) | 175 (170; 180) | 175 (170; 180) | 175 (170; 180) | 174 (167; 180) | 0.001 |
| Weight, kg, median (IQR) | 80 (73; 90) | 84 (75; 92) | 81 (75; 93) | 78 (69; 88) | 0.000 |
| BMI, kg/m^2^, median (IQR) | 26.3 (24.1; 29.7) | 27.0 (24.7; 30.0) | 26.6 (24.2; 30.1) | 25.2 (22.9; 28.1) | 0.000 |
| **Chronic comorbidities** |  |  |  |  |  |
| Hypertension, yes, n (%) | 504 (44.8) | 63 (47.7) | 365 (46.9) | 76 (35.5) | 0.009 |
| Diabetes mellitus, yes, n (%) | 266 (18.8) | 35 (21.2) | 1818 (18.3) | 50 (19.0) | 0.667 |
| Myocardial infarction, yes, n (%) | 230 (27.3) | 31 (31.0) | 163 (28.5) | 36 (20.9) | 0.099 |
| Percutaneous coronary intervention, yes, n (%) | 210 (25.3) | 28 (28.9) | 146 (26.1) | 36 (20.7) | 0.246 |
| Coronary artery bypass graft, yes, n (%) | 112 (15.3) | 16 (18.8) | 79 (16.1) | 17 (11.0) | 0.194 |
| Heart failure, yes, n (%) | 145 (19.0) | 20 (22.5) | 104 (20.2) | 21 (13.2) | 0.098 |
| Charlson comorbidity index, points, median (IQR) | 4.0 (2.0; 5.0) | 4.0 (3; 6.0) | 4.0 (2.0; 5.0) | 4.0 (2.0; 5.0) | 0.160 |
| **Pre-hospital setting / interventions** |  |  |  |  |  |
| Location of cardiac arrest, n (%) |  |  |  |  |  |
| Home | 741 (52.3) | 86 (52.1) | 505 (51.0) | 150 (57.0) |  |
| Public place | 509 (35.9) | 58 (35.2) | 372 (37.6) | 79 (30.0) |  |
| Other | 168 (11.8) | 21 (12.7) | 113 (11.4) | 34 (12.9) | 0.259 |
| Witnessed cardiac arrest, yes, n (%) | 1295 (91.3) | 155 (93.9) | 899 (90.8) | 241 (91.6) | 0.409 |
| Bystander performed CPR, yes, n (%) | 1148 (81.0) | 132 (80.0) | 810 (81.8) | 206 (78.3) | 0.416 |
| Type of rhythm, n (%) |  |  |  |  |  |
| Not shockable | 390 (27.5) | 55 (33.3) | 266 (26.9) | 69 (26.2) |  |
| Shockable | 1028 (72.5) | 110 (66.7) | 724 (73.1) | 194 (73.8) | 0.199 |
| Time to ROSC, minutes, median (IQR) | 25.0 (17.0; 39.0) | 26.0 (18.5; 40.0) | 25.0 (17.0; 40.0) | 25.0 (15.0; 34.0) | 0.024 |
| TTM randomization treatment, n (%) |  |  |  |  |  |
| Normothermia | 712 (50.2) | 88 (53.3) | 496 (50.1) | 128 (48.7) |  |
| Hypothermia | 706 (49.8) | 77 (46.7) | 494 (49.9) | 135 (51.3) | 0.638 |

**Table S5. Patients’ clinical outcomes in the overall population and stratified according to the new oxygen thresholds.** Data are expressed as median (interquartile range, IQR). ICU, intensive care unit; STEMI, ST elevation myocardial infarction; LOS, length of stay; mRS, modified Rankin Scale; mRS binary defined as poor outcome if points 4-6, good outcome if points 1-3.

|  | **Overall**  **(n=1418, 100.0%)** | **PaO_2_ <69 mmHg**  **(n=165, 11.6%)** | **PaO_2_ 69-195 mmHg**  **(n=990, 69.8%)** | **PaO_2_ >195 mmHg**  **(n=263, 18.5%)** | **p-**  **value** |
| --- | --- | --- | --- | --- | --- |
| **Neurologic outcome** |  |  |  |  |  |
| mRS at 6 months, points, median (IQR) | 6.0 (1.0; 6.0) | 6.0 (2.0; 6.0) | 6.0 (1.0; 6.0) | 6.0 (2.0; 6.0) | 0.192 |
| mRS poor outcome (4-6), n (%) | 740 (55.9) | 95 (62.1) | 508 (54.9) | 137 (55.9) | 0.254 |
| **Hospital / ICU admission** |  |  |  |  |  |
| Shock diagnosis on admission, yes, n (%) | 424 (29.9) | 65 (39.4) | 294 (29.7) | 65 (24.7) | 0.005 |
| STEMI diagnosis on admission, yes, n (%) | 553 (39.4) | 62 (39.0) | 396 (40.2) | 95 (36.7) | 0.583 |
| ICU LOS, days, median (IQR) | 5.1 (3.0; 8.7) | 5.0 (2.9; 8.8) | 5.1 (3.0; 8.9) | 4.8 (3.0; 7.4) | 0.166 |
| Hospital LOS, days, median (IQR) | 9.5 (5.0; 17.0) | 8.0 (3.0; 16.8) | 10.0 (5.0; 18.0) | 9.0 (5.0; 14.0) | 0.036 |
| Time to extubation, days, median (IQR) | 3.6 (1.9; 6.0) | 3.7 (2.0; 5.9) | 3.8 (1.9; 6.5) | 3.3 (1.9; 5.1) | 0.288 |
| Ventilator free days at 30 days, days, median (IQR) | 26.4 (23.8; 28.1) | 26.3 (24.1; 28.0) | 26.3 (23.5; 28.1) | 26.7 (24.8; 28.1) | 0.396 |
| Ventilator free days before ICU discharge, days, median (IQR) | 1.0 (0.0; 2.0) | 0.0 (0.0; 2.0) | 1.0 (0.0; 2.0) | 1.0 (0.0; 2.0) | 0.511 |
| Discharged alive from ICU, yes, n (%) | 893 (63.0) | 92 (55.8) | 637 (634.3) | 164 (62.4) | 0.104 |
| Discharged alive from Hospital, n (%) | 766 (54.0) | 77 (46.7) | 550 (55.6) | 139 /52.9) | 0.097 |

**Table S6. Ventilatory setting, respiratory mechanics and gas exchange at ICU admission stratified according to the new oxygen thresholds.** Table legend: Data are expressed as median (interquartile range, IQR). PBW, predicted body weight; PaO_2_, arterial partial pressure of oxygen; PaCO_2_, arterial partial pressure of carbon dioxide; FiO_2_, fraction of inspired oxygen. Ventilatory ratio was calculated as = (minute ventilation × PaCO_2_) / (PBW × 100 × 37.5).

|  | **Overall**  **(n=1418, 100.0%)** | **PaO_2_**  **<69 mmHg**  **(n=165, 11.6%)** | **PaO_2_**  **69-195 mmHg**  **(n=990, 69.8%)** | **PaO_2_**  **>195 mmHg**  **(n=263, 18.5%)** | **p-**  **value** |
| --- | --- | --- | --- | --- | --- |
| **Ventilatory setting and pulmonary mechanics at ICU admission** |  |  |  |  |  |
| Respiratory rate, breaths/min, median (IQR) | 16.0 (14.0; 20.0) | 16.0 (15.0; 20.0) | 16.0 (15.0; 20.0) | 16.0 (14.0; 18.0) | 0.010 |
| Positive end-expiratory pressure, cmH_2_O, median (IQR) | 6.0 (5.0; 8.0) | 7.0 (5.0; 10.0) | 6.0 (5.0; 8.0) | 6.0 (5.0; 8.0) | 0.010 |
| Plateau pressure, cmH_2_O, median (IQR) | 20.0 (17.0; 24.0) | 22.0 (19.0; 25.0) | 20.0 (17.0; 23.0) | 20.0 (16.0; 22.5) | 0.004 |
| Tidal volume, mL, median (IQR) | 500 (445; 560) | 500 (437; 582) | 500 (444; 559) | 500 (450; 560) | 0.919 |
| Tidal volume, mL/kg PBW, median (IQR) | 7.2 (6.4; 8.2) | 7.3 (6.4; 8.6) | 7.1 (6.3; 8.1) | 7.6 (6.6; 8.5) | 0.022 |
| Driving pressure, cmH_2_O, median (IQR) | 13.0 (10.0; 16.0) | 14.0 (12.0; 16.0) | 13.0 (10.0; 16.0) | 12.0 (10.0; 16.0) | 0.142 |
| Compliance of the respiratory system, mL/cmH_2_O, median (IQR) | 40.0 (31.0; 50.0) | 36.0 (30.0; 46.0) | 40.0 (31.0; 50.0) | 42.0 (32.0; 53.0) | 0.071 |
| Mechanical Power, j/min, median (IQR) | 16.2 (12.7; 21.6) | 18.3 (14.4; 22.9) | 16.0 (12.5; 21.5) | 15.2 (11.5; 20.6) | 0.006 |
| Ventilatory ratio, median (IQR) | 1.45 (1.16; 1.83) | 1.60 (1.24; 2.18) | 1.46 (1.18; 1.81) | 1.32 (1.03; 1.75) | 0.001 |
| **Gas exchange** |  |  |  |  |  |
| Fraction of inspired oxygen (FiO_2_), %, median (IQR) | 60 (50; 88) | 70 (50; 100) | 60 (46; 80) | 70 (50; 100) | 0.000 |
| PaO_2_, mmHg, median (IQR) | 108.7 (83.2; 163.0) | 60.0 (51.7; 65.2) | 105.0 (87.0; 133.0) | 273.0 (231.7; 342.7) | 0.000 |
| PaO_2_/ FiO_2_ ratio, mmHg, median (IQR) | 195.0 (122.8; 307.3) | 75.0 (57.6; 113.6) | 180.0 (130.0; 256.9) | 410.0 (318.4; 521.9) | 0.000 |
| PaCO_2_, mmHg, median (IQR) | 45.7 (39.7; 53.2) | 49.5 (41.2; 59.2) | 46.0 (40.5; 53.2) | 42.0 (36.7; 49.2) | 0.000 |
| pHa, median (IQR) | 7.3 (7.2; 7.3) | 7.2 (7.1; 7.3) | 7.3 (7.2; 7.3) | 7.3 (7.2; 7.3) | 0.001 |
| Base excess, mEq/L, median (IQR) | -6.5 (-10.0; -3.7) | -7.3 (-10.1; -4.5) | -6.2 (-9.7; -3.5) | -6.6 (-10.5; -4.0) | 0.204 |

**Figure S1. Hourly oxygen trajectories in the overall population, and by survival status (survivors and non survivors).** Partial pressure of oxygen (PaO_2_) trajectories over the first 72-h after ICU admission are summarized as medians. PaO_2_ values decreased significantly during the first 40 hours and then leveled-off afterwards with median values ranging from 80 to 85 mmHg. shows the frequency distribution of the episodes of PaO_2_ <60 mmHg and > 300 mmHg in the whole population during the first 72 hours of mechanical ventilation.

**Figure S2 a. Kaplan-Meier curve considering conventional and new thresholds (mmHg). Most deaths occurred during the first 30 days of the follow-up.** The log-rank test p-value did not achieve significance (p = 0.140).


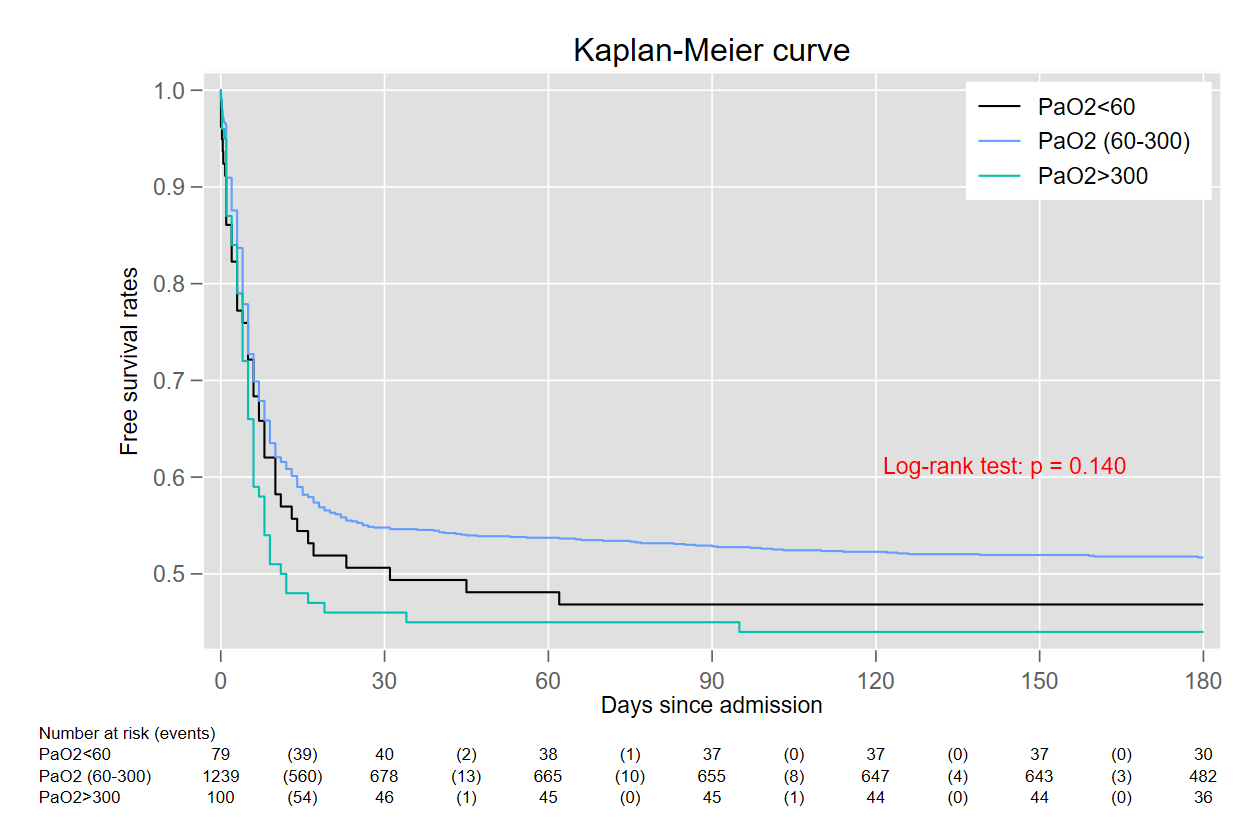


**Figure S3. Hypoxemia and hyperoxemia mortality risk difference considering the dose of oxygen (exposure over time, area under the curve, PaO_2_-AUC).** Repeated measures of PaO_2_ were considered as a single time point reflecting the cumulative effect over time of PaO_2_. PaO_2_-AUC represents the numerical integration of PaO_2_ values and the time between measurements, and as such, provides the cumulative effect over time of PaO_2_. The higher/lower the value of PaO_2_, indeed influence the AUC in that direction; however, it also includes the time interval between measurements as a function. The narrower is the interval, the higher is the AUC, independently of PaO_2_ raw values, or vice versa. We tested the interaction between PaO_2_ categorical levels using traditional cut-points (hypo/normo/hyperoxemia) with the PaO_2_-AUC and determining the risk difference among hypo vs normoxemia and hyperoxemia vs normoxemia along the PaO_2_ -AUC continuum. Left and right panel depicts the differences in mortality risk (expressed as Hazard ratios, HRs) between PaO_2_-AUC in the hypoxemia and hyperoxemia groups, respectively with normoxemia as a reference category. In this Cox regression, PaO_2_-AUC was modelled with a fractional polynomial of second-degree FP [1 1], and included the following covariates: TTM2 randomization group, age (years), cardiac arrest witnessed, ROSC (min), bystander performed CPR, shockable rhythm, cardiac arrest location (home, public place, other), shock diagnosis on admission, and STEMI diagnosis on admission.


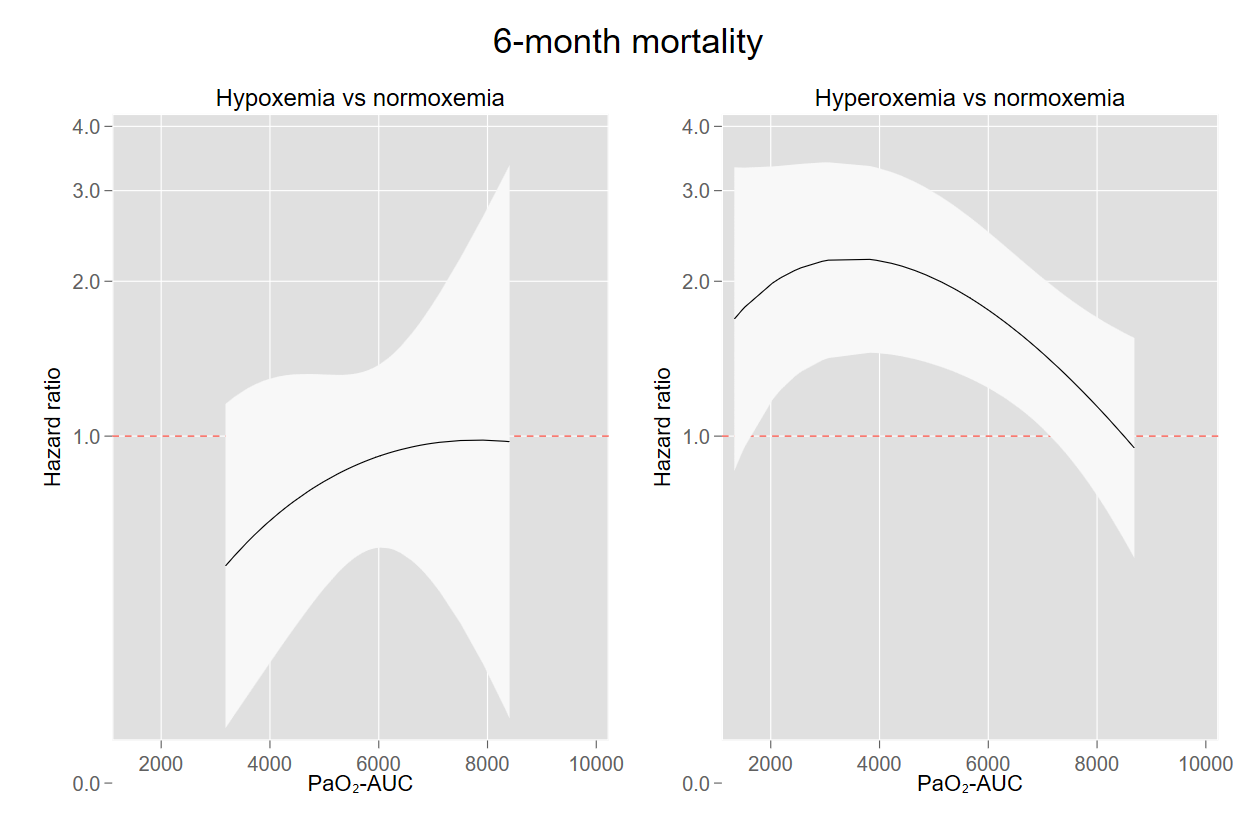


**Figure S4. Interaction between PaO_2_ (continuous) and TTM2-arms.** Difference in mortality risk (expressed as HRs - interaction p-value = 0.9974) for hypothermia (with normothermia as a reference category) with estimates listed for each value of PaO_2_. We tested the interaction between PaO2 categorical levels using Hypo/normothermia as the interacting factor. In this Cox regression, PaO_2_ was modelled with a fractional polynomial of second-degree FP [1 1], and included the following covariates: TTM2 randomization group, age (years), cardiac arrest witnessed, time to return to spontaneous circulation, ROSC (min), bystander performed cardiopulmonary resuscitation, CPR, shockable rhythm, cardiac arrest location (home, public place, other), shock diagnosis on admission, and ST-elevated myocardial infarction, STEMI diagnosis on admission.

**Figure S5. Frequency distribution of modified Rankin Score (mRS) among partial pressure of oxygen, PaO_2_ classes.**


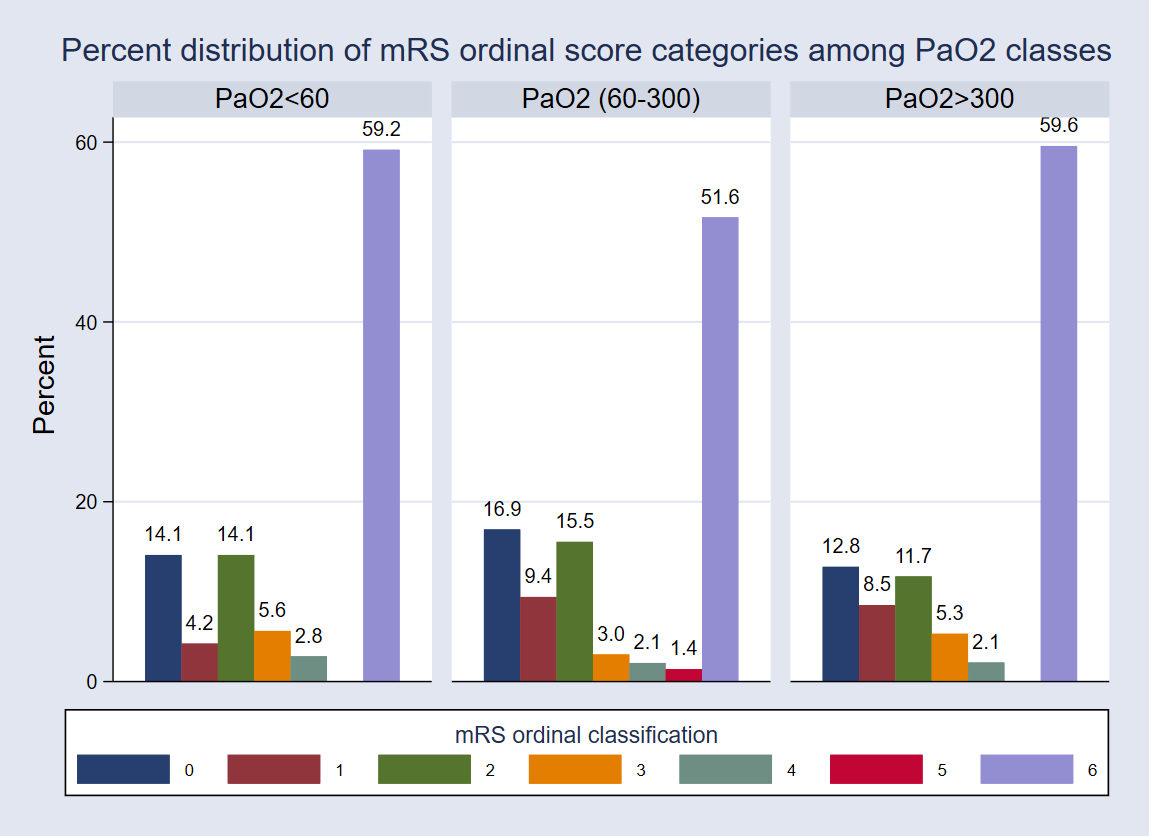


## Figure S6. Predicted probability of achieving a modified Rankin Score (mRS) 4 or 5 according to PaO_2_ values. Left and right panel of this figure depicts the predicted probability of achieving a mRS score 4 and 5, respectively, according to PaO_2_ values. Considering only mRS score 4 and 5, no significant differences among PaO_2_ classes were observed (the estimates for score 4 were: hypoxemia vs normoxemia, probability = +0.0005; p=0.59 and hyperoxemia vs normoxemia, probability = −0.0003; p=0.79. The estimates for mRS score 5 were: hypoxemia vs normoxemia, probability = +0.0002; p=0.62 and hyperoxemia vs normoxemia, probability = −0.00017; p=0.79). Similarly, modeling PaO_2_ as continuous we found no association between PaO_2_ and mRS score 4 (left panel) and score 5 (right panel of ESM figure S6).

**Figure S7. Analysis for the definition of the best cut off point of oxygen values for the prediction of poor neurological outcome.**

ESM Appendix. Sensitivity analysis modelling PaO_2_ categorical using a90 mmHg as cut-point of hyperoxemia, in a Cox regression for 6-m mortality and using the same set of covariates as the main model. Hyperoxemia did not achieve statistical significance as compare with normoxemia group.

Comparison of baseline characteristics/ventilatory markers/outcomes among those with and without missing values in PaO_2_, as an indirect approach to uncover a potential selection bias. No significative difference in outcomes and characteristics was found. Data are reported as mean (standard deviation, SD) and number (percentage, %). Legend: n=number of patients, BMI, body mass index, IBW, ideal body weight, ROSC, return of spontaneous circulation, CPR, cardio-pulmonary resuscitation, PaO2,partial pressure of oxygen, Hx, history, TTM, target temperature management.

| **Indicator for PaO2 missingness** | **PaO2 complete** | **PaO2 missing** | **P-value** |
| --- | --- | --- | --- |
| n (%) | 1418 (76.2) | 443 (23.8) |  |
|  |  |  |  |
|  |  |  |  |
| **Baseline patient characteristics ************ | ************** | ************** | ************** |
| Age, years, mean (SD) | 64 (14) | 64 (13) | 0.943 |
|  |  |  |  |
| Categories of PaO2, n (%) |  |  |  |
| <30 | 30 (2.1) | 10 (2.3) |  |
| 30-49 | 185 (13.0) | 49 (11.1) |  |
| 50-69 | 656 (46.3) | 223 (50.3) |  |
| >= 70 | 547 (38.6) | 161 (36.3) | 0.437 |
|  |  |  |  |
| Gender, n (%) |  |  |  |
| Male | 1126 (79.4) | 351 (79.2) |  |
| Female | 292 (20.6) | 92 (20.8) | 0.937 |
|  |  |  |  |
| Height, cm, mean (SD) | 175 (9) | 174 (10) | 0.204 |
|  |  |  |  |
| Weight, Kg, mean (SD) | 83 (17) | 83 (18) | 0.995 |
|  |  |  |  |
| BMI - original variable, mean (SD) | 27.4 (5.8) | 27.6 (5.7) | 0.477 |
|  |  |  |  |
| Categories of BMI, n (%) |  |  |  |
| 1. Severe thinness | 8 (0.6) | 1 (0.2) |  |
| 2. Moderate thinness | 8 (0.6) | 1 (0.2) |  |
| 3. Mild thinness | 8 (0.6) | 5 (1.2) |  |
| 4. Normal range | 491 (35.5) | 137 (32.4) |  |
| 5. Pre-obese | 535 (38.7) | 175 (41.4) |  |
| 6. Obese class I | 224 (16.2) | 66 (15.6) |  |
| 7. Obese class II | 71 (5.1) | 25 (5.9) |  |
| 8. Obese class III | 39 (2.8) | 13 (3.1) | 0.639 |
|  |  |  |  |
| Ideal body weight, mean (SD) | 69 (9) | 69 (10) | 0.254 |
|  |  |  |  |
| Prior Hx. of hypertension, n (%) |  |  |  |
| No | 620 (55.2) | 203 (59.4) |  |
| Yes | 504 (44.8) | 139 (40.6) | 0.171 |
|  |  |  |  |
| Prior Hx. of diabetes, n (%) |  |  |  |
| No | 1152 (81.2) | 369 (83.3) |  |
| Yes | 266 (18.8) | 74 (16.7) | 0.329 |
|  |  |  |  |
| Prior Hx. of MI, n (%) |  |  |  |
| No | 614 (72.7) | 201 (76.1) |  |
| Yes | 230 (27.3) | 63 (23.9) | 0.276 |
|  |  |  |  |
| Prior Hx. of PCI, n (%) |  |  |  |
| No | 620 (74.7) | 203 (77.2) |  |
| Yes | 210 (25.3) | 60 (22.8) | 0.415 |
|  |  |  |  |
| Prior Hx. of CABG, n (%) |  |  |  |
| No | 620 (84.7) | 203 (84.6) |  |
| Yes | 112 (15.3) | 37 (15.4) | 0.965 |
|  |  |  |  |
| Hx. of heart failure, n (%) |  |  |  |
| No | 618 (81.0) | 198 (83.9) |  |
| Yes | 145 (19.0) | 38 (16.1) | 0.314 |
|  |  |  |  |
| Charlson comorbidity index, median (IQR) | 4.00 (2.00; 5.00) | 4.00 (2.00; 5.00) | 0.176 |
|  |  |  |  |
|  |  |  |  |
|  |  |  |  |
| **Pre-hospital settings/interventions ************ | ************** | ************** | ************** |
| Location of cardiac arrest, n (%) |  |  |  |
| 1. Home | 741 (52.3) | 237 (53.5) |  |
| 2. Other | 168 (11.8) | 57 (12.9) |  |
| 3. Public place | 509 (35.9) | 149 (33.6) | 0.645 |
|  |  |  |  |
| Was cardiac arrest witnessed?, n (%) |  |  |  |
| No | 123 (8.7) | 36 (8.1) |  |
| Yes | 1295 (91.3) | 407 (91.9) | 0.719 |
|  |  |  |  |
| Bystander performed CPR, n (%) |  |  |  |
| No | 270 (19.0) | 104 (23.5) |  |
| Yes | 1148 (81.0) | 339 (76.5) | 0.052 |
|  |  |  |  |
| Type of rhytm, n (%) |  |  |  |
| not shockable | 390 (27.5) | 100 (22.6) |  |
| shockable | 1028 (72.5) | 343 (77.4) | 0.040 |
|  |  |  |  |
| Time to return of spontaneous circulation (ROSC), min, median (IQR) | 25.0 (17.0; 39.0) | 27.0 (16.0; 42.3) | 0.281 |
|  |  |  |  |
|  |  |  |  |
| **Previous RCT ************ | ************** | ************** | ************** |
| TTM2: randomization treatment, n (%) |  |  |  |
| Normothermia | 712 (50.2) | 219 (49.4) |  |
| Hypothermia | 706 (49.8) | 224 (50.6) | 0.776 |
|  |  |  |  |
| tympanic temperature at admission, centigrades, mean (SD) | 35.4 (1.1) | 35.3 (1.1) | 0.265 |
|  |  |  |  |
|  |  |  |  |
| **Modified Rankin Scale (mRS) at 6 months ************ | ************** | ************** | ************** |
| Modified Rankin Scale (mRS): scale defining the neurological outcome at 6 months, median (IQR) | 6.00 (1.00; 6.00) | 6.00 (1.00; 6.00) | 0.570 |
|  |  |  |  |
| Modified Rankin Scale (mRS): scale defining the neurological outcome at 6 months, n (%) |  |  |  |
| 0 | 218 (16.5) | 70 (16.5) |  |
| 1 | 120 (9.1) | 47 (11.1) |  |
| 2 | 201 (15.2) | 58 (13.7) |  |
| 3 | 44 (3.3) | 22 (5.2) |  |
| 4 | 28 (2.1) | 8 (1.9) |  |
| 5 | 16 (1.2) | 4 (0.9) |  |
| 6 | 696 (52.6) | 215 (50.7) | 0.504 |
|  |  |  |  |
| mRS binary: poor outcome (4-6) and good outcome (1-3), n (%) |  |  |  |
| Good | 583 (44.1) | 197 (46.5) |  |
| Poor | 740 (55.9) | 227 (53.5) | 0.388 |
|  |  |  |  |
|  |  |  |  |
| **Hospital/ICU admission ************ | ************** | ************** | ************** |
| Shock diagnosis on admission, n (%) |  |  |  |
| FALSE | 994 (70.1) | 331 (74.7) |  |
| TRUE | 424 (29.9) | 112 (25.3) | 0.061 |
|  |  |  |  |
| STEMI diagnosis on admission, n (%) |  |  |  |
| No | 850 (60.6) | 240 (55.0) |  |
| Yes | 553 (39.4) | 196 (45.0) | 0.040 |
|  |  |  |  |
| Hospital LOS, days, median (IQR) | 9.5 (5.0; 17.0) | 10.0 (4.0; 17.5) | 0.495 |
|  |  |  |  |
| Was the patient mechanically ventilated?, n (%) |  |  |  |
| No | 7 (0.5) | 5 (1.1) |  |
| Yes | 1411 (99.5) | 438 (98.9) | 0.145 |
|  |  |  |  |
| Ventilator free days before ICU discharge, median (IQR) | 1.00 (0.00; 2.00) | 1.00 (0.00; 2.00) | 0.904 |
|  |  |  |  |
| Was the patient discharged alive from the ICU?, n (%) |  |  |  |
| No | 525 (37.0) | 177 (40.0) |  |
| Yes | 893 (63.0) | 266 (60.0) | 0.267 |
|  |  |  |  |
| Was the patient discharged alive from the hospital?, n (%) |  |  |  |
| No | 652 (46.0) | 207 (46.7) |  |
| Yes | 766 (54.0) | 236 (53.3) | 0.783 |
|  |  |  |  |
| Survival status since hosp/ICU admission, n (%) |  |  |  |
| Alive | 722 (50.9) | 228 (53.0) |  |
| Died | 696 (49.1) | 202 (47.0) | 0.444 |
|  |  |  |  |
| Days to death/censored up to 180 days (end of study), median (IQR) | 168 (5; 187) | 167 (4; 187) | 0.233 |

| **Indicator for PaO2 missingness** | **PaO2 complete** | **PaO2 missing** | **P-value** |
| --- | --- | --- | --- |
| n (%) | 1418 (76.2) | 443 (23.8) |  |
|  |  |  |  |
|  |  |  |  |
| **Ventilatory markers at ICU admission ************ | ************** | ************** | ************** |
| Positive end-expiratory pressure, cmH{subscript:2}O, median (IQR) | 6.00 (5.00; 8.00) | 6.00 (5.00; 8.00) | 0.451 |
|  |  |  |  |
| Tidal Volume, (ml*kg−1 per PBW), mean (sd) | 7.59 (5.76) | 7.02 (1.72) | 0.249 |
|  |  |  |  |
| Driving pressure, cmH{subscript:2}O, median (IQR) | 13.0 (10.0; 16.0) | 12.0 (10.0; 16.0) | 0.288 |
|  |  |  |  |
| Compliance of respiratory system, median (IQR) | 40 (31; 50) | 39 (26; 51) | 0.521 |
|  |  |  |  |
| Mechanical power, j/min, median (IQR) | 16.2 (12.7; 21.6) | 16.6 (12.1; 22.3) | 0.950 |
|  |  |  |  |
| Ventilator ratio at baseline, median (IQR) | 1.45 (1.16; 1.83) | 2.19 (1.53; 3.11) | 0.051 |
|  |  |  |  |
| fraction of inspired O2, %, median (IQR) | 60 (50; 88) | 60 (45; 90) | 0.505 |
|  |  |  |  |
| Partial pressure of carbon dioxide (PaCO{subscript:2}), mmHg, median (IQR) | 45.7 (39.7; 53.2) | 48.8 (39.5; 51.9) | 0.665 |
|  |  |  |  |
| pH, mean (SD) | 7.24 (0.12) | 7.22 (0.11) | 0.446 |
|  |  |  |  |
| Base excess, mEq/L, median (IQR) | -6.5 (-10.0; -3.7) | -7.2 (-9.2; -5.1) | 0.549 |
